# Molecular time machines unleashed: small-molecule-driven reprogramming to reverse the senescence

**DOI:** 10.1093/stcltm/szaf069

**Published:** 2026-01-12

**Authors:** Chunyin Tang, Zhen Zhang, Chunsong Yang, Luxin Li, Jie Li, Xuejiao Cheng, Wei Zhou, Yunzhu Lin, Linan Zeng, Lingli Zhang

**Affiliations:** Department of Pharmacy/Evidence-Based Pharmacy Center, West China Second University Hospital, Sichuan University, Chengdu, China; Children’s Medicine Key Laboratory of Sichuan Province, Chengdu, China; Key Laboratory of Birth Defects and Related Diseases of Women and Children, Sichuan University, Ministry of Education, Chengdu, China; Heilongjiang Key Laboratory of Anti-Fibrosis Biotherapy, Mudanjiang Medical University, Mudanjiang, China; Department of Pharmacy/Evidence-Based Pharmacy Center, West China Second University Hospital, Sichuan University, Chengdu, China; Children’s Medicine Key Laboratory of Sichuan Province, Chengdu, China; Key Laboratory of Birth Defects and Related Diseases of Women and Children, Sichuan University, Ministry of Education, Chengdu, China; Heilongjiang Key Laboratory of Anti-Fibrosis Biotherapy, Mudanjiang Medical University, Mudanjiang, China; Department of Pharmacy/Evidence-Based Pharmacy Center, West China Second University Hospital, Sichuan University, Chengdu, China; Children’s Medicine Key Laboratory of Sichuan Province, Chengdu, China; Department of Pharmacy/Evidence-Based Pharmacy Center, West China Second University Hospital, Sichuan University, Chengdu, China; Children’s Medicine Key Laboratory of Sichuan Province, Chengdu, China; Department of Pharmacy/Evidence-Based Pharmacy Center, West China Second University Hospital, Sichuan University, Chengdu, China; Children’s Medicine Key Laboratory of Sichuan Province, Chengdu, China; Department of Pharmacy/Evidence-Based Pharmacy Center, West China Second University Hospital, Sichuan University, Chengdu, China; Children’s Medicine Key Laboratory of Sichuan Province, Chengdu, China; Key Laboratory of Birth Defects and Related Diseases of Women and Children, Sichuan University, Ministry of Education, Chengdu, China; Department of Pharmacy/Evidence-Based Pharmacy Center, West China Second University Hospital, Sichuan University, Chengdu, China; Children’s Medicine Key Laboratory of Sichuan Province, Chengdu, China; Key Laboratory of Birth Defects and Related Diseases of Women and Children, Sichuan University, Ministry of Education, Chengdu, China; NMPA Key Laboratory for Technical Research on Drug Products In Vitro and In Vivo Correlation, Chengdu, China; West China Biomedical Big Data Center, West China Hospital, Sichuan University, Chengdu, China; Department of Pharmacy/Evidence-Based Pharmacy Center, West China Second University Hospital, Sichuan University, Chengdu, China; Children’s Medicine Key Laboratory of Sichuan Province, Chengdu, China; Key Laboratory of Birth Defects and Related Diseases of Women and Children, Sichuan University, Ministry of Education, Chengdu, China; NMPA Key Laboratory for Technical Research on Drug Products In Vitro and In Vivo Correlation, Chengdu, China; Chinese Evidence-based Medicine Center, West China Hospital, Sichuan University, Chengdu, China; West China Biomedical Big Data Center, West China Hospital, Sichuan University, Chengdu, China

**Keywords:** small molecules, cell reprogramming, anti-senescence, chemical reprogramming, pluripotent stem cell

## Abstract

Cellular reprogramming, a method of “resetting” the epigenetic clock by reversing the differentiation state of cells, has emerged as a promising approach to anti-aging, offering new strategies to slow down the aging process. Researchers convert differentiated cells into a pluripotent stem cell state through transcription factors or chemicals, restoring cellular youthfulness and regenerative capacity. This technology holds potential for tissue repair, lifespan extension, organ function improvement, and treatment of age-related diseases. In addition, cell reprogramming provides a novel pathway for disease modeling and drug screening, potentially accelerating the development and clinical application of anti-aging drugs. However, it faces challenges including safety, efficiency, and ethical considerations. This article focuses on the prospects of small-molecule-induced cell reprogramming for anti-aging, covering its mechanisms, applications, current limitations, and future directions to facilitate clinical translation and breakthroughs in human healthspan extension.

## Graphical abstract

**Graphical Abstract:**
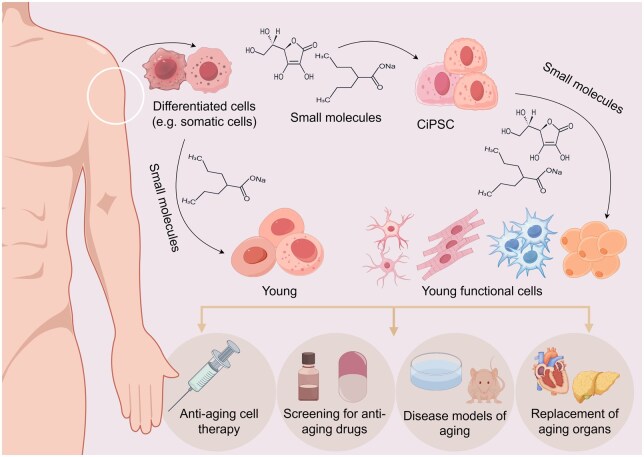

Significance statement Small-molecule cocktails reset epigenetic aging clocks and reprogram cells into pluripotent stem cells, enabling partial rejuvenation without genetic manipulation. These compounds selectively target senescence-associated hallmarks, offering scalable and tunable strategies for anti-aging cell therapy, anti-aging drug screening, building aging models, and replacing aging organs to extend healthy lifespan.

## Introduction

Cell reprogramming refers to the process of altering the epigenetic state and gene expression pattern of cells by human intervention, transforming them from one differentiation state to another, or even reversing them to pluripotency or totipotency.^1^^,^^2^ This process challenges the traditional developmental biology dogma of “irreversible cell fate” and demonstrates the plasticity of cell fate. Historically, it was widely believed that cellular differentiation during development was unidirectional, with mature cells being incapable of returning to a pluripotent stem cell state. Cell biologists generally believed that differentiated cells retained only the genes necessary for maintaining their identity and function and that reversion to an undifferentiated state was impossible. A paradigm shift occurred in 1952, when Robert Briggs and Thomas J King successfully transplanted nuclei from frog blastocyst-stage cells into enucleated oocytes and found that some of the nuclear-transplanted embryos could develop into tadpoles, indicating that the nuclei of blastocyst stage cells still retained their developmental potentials.^3^ This finding was further substantiated in 1962 by John Gurdon, achieved the cloning of Xenopus laevis that developed to adulthood by transplanting nuclei from tadpole intestinal epithelial cells into enucleated oocytes using micromanipulation techniques.^4^ The study demonstrated for the first time that the nucleus of differentiated cells retains an intact genome and can be reprogrammed to restore totipotency through the oocyte cytoplasmic environment, laying the theoretical foundation for cellular reprogramming. Subsequently, in 1987 and 1991, Harold Weintraub’s team identified the pivotal role of the *MyoD* gene family in muscle cell differentiation. Their work systematically demonstrated that *MyoD*, as a master regulator, could directly convert fibroblasts into skeletal muscle cells.^5^ This breakthrough provided the first evidence that a single factor could drive cell fate conversion, marking the inception of the “direct reprogramming” concept. The birth of Dolly the sheep clone in 1996 demonstrated that the nuclei of highly differentiated somatic cells can still be reprogrammed to a state of totipotency to support embryonic development to adulthood.^6^^,^^7^ The *Science* magazine named the cloning of Dolly the Sheep as one of the world’s top 10 scientific breakthroughs of 1997. Until 2006, a team of Japanese scientists led by Shinya Yamanaka made a breakthrough in reprogramming mouse fibroblasts into induced pluripotent stem cells (iPSCs) using four transcription factors (TFs), namely *Oct4, Sox2, Klf4*, and *c-Myc* (OSKM), which marked the first successful complete reprogramming and established the term “cellular reprogramming.”^8^ After 2010, reprogramming technologies expanded from TFs to chemical small-molecule induction (such as vitamin C and VPA).^9^ In addition, the continuous innovation of TFs-based reprogramming methods, including viral vector, CRISPR-dCas9 system, and mRNA-induced reprogramming,^10^^,^^11^ has promoted the development of safer, more precise, and more diversified approaches.

**Figure 1.**
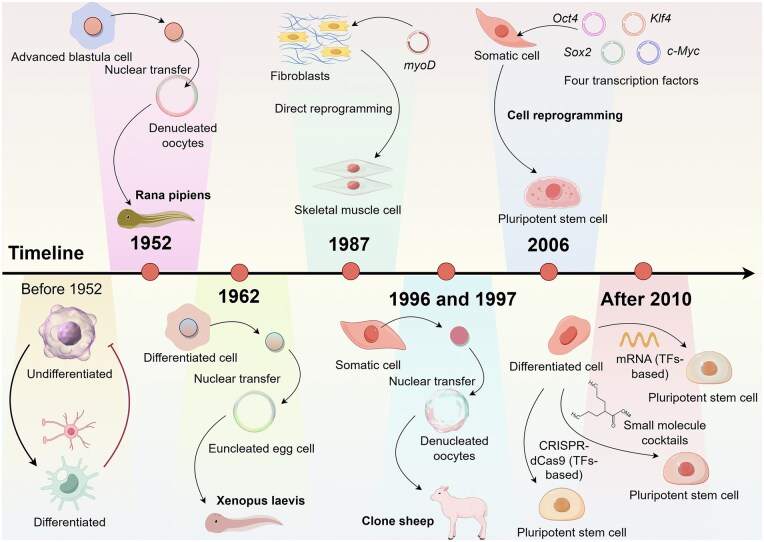
The timeline for the progression of cell reprogramming.

Therefore, the core mechanism of cellular reprogramming is to reset the developmental clock of cells by regulating key TFs, epigenetic modifications, or signaling pathways, thereby conferring new functions or repairing the senescent/damaged state of cells. Figure 1 briefly illustrates these advances in cell reprogramming.

Aging is a multifactorial biological process encompassing the combined effects of genetic, environmental, metabolic, and immunological changes.^12–16^ These alterations lead to a gradual decline in the functioning of the organism and an increased susceptibility to a wide range of diseases, such as neurodegenerative disorders, cardiovascular diseases, and cancer.^17^^,^^18^ Recent studies have shown that aging exhibits 12 major characteristics: genomic instability (e.g., genetic mutation), telomere shortening (e.g., DNA easily damaged), epigenetic changes (e.g., DNA methylation clock shift and histone modification disorder), unbalance of proteostasis (e.g., accumulation of misfolded proteins), disabled macroautophagy (e.g., decreased hydrolysis of intracellular substances and reduced removal of specific substrates), deregulated nutrient-sensing (e.g., abnormalities in mTOR and AMPK pathways), mitochondrial disorder (e.g., ATP production decreases and ROS increases), cellular senescence (e.g., elevated secretion of SASP-type factors), stem cell depletion (e.g., reduced regenerative capacity of hematopoietic stem cells), altered intercellular communication (e.g., paracrine communication disorders and autocrine regulatory failure), persistent inflammation (e.g., inflammatory cytokine storm), and dysbiosis (e.g., imbalance of gut microbiota).^19^ Each feature elucidates the causes and processes of aging from different perspectives. Understanding these mechanisms provides a theoretical basis for the development of anti-aging and aging-related diseases’ intervention strategies.

Senescence is an inevitable outcome of cellular development driven by time, while cell reprogramming represents the reverse process of cellular development, but not merely a simple reversal. Nevertheless, cellular reprogramming can partially reverse the molecular features of aging and provide a biological basis for the treatment of aging and age-related diseases. Our study summarizes advances in small molecule compounds that induce cellular reprogramming for anti-aging and age-related disease therapies to promote their early clinical application.

## Cell reprogramming techniques: from degeneration to rejuvenation

Cell reprogramming reverses mature cells to pluripotency or even directly rejuvenates aged cells by remodeling epigenetics and gene networks.^20^ Breakthroughs in its core mechanisms and novel technological pathways continue to refresh the cognition of cell age plasticity. The molecular mechanisms of cell reprogramming to reverse aging mainly involve epigenetic remodeling, regulation of telomere dynamics, mitochondrial remodeling, reconstitution of protein homeostasis, and reshaping of the inflammatory microenvironment.^21–25^ There are also dynamic intersections and synergies between these mechanisms. Table S1, see online supplementary material for a color version of this figure, gathered the current reprogramming techniques for anti-aging, highlighting their pros and cons.

## Cell reprogramming induced by small-molecule compounds for anti-aging

Traditional transcription factor–mediated reprogramming (e.g., Yamanaka factor OSKM) reverses cellular senescence but carries a risk of tumorigenicity and technical complexity. Small molecules have become promising tools in reversing cellular senescence with their unique advantages. Most of the small-molecule compounds affect cell fate by epigenetic regulators, metabolic regulators, signaling regulators, and cell cycle regulators. Table 1 aggregates representative small-molecule compounds that can highly drive cell reprogramming. Interestingly, certain compounds could also be employed in anti-aging via these mechanisms of action.

**Table 1.** Representative small-molecule compounds for cell reprogramming. ChemSpider ID comes from the https://www.chemspider.com website.

Term	Chemical formula	Mechanism	Function	Function classification	ChemSpider ID	References
**Epigenetic modulator**						
**5-Azacytidine(5-aza)**	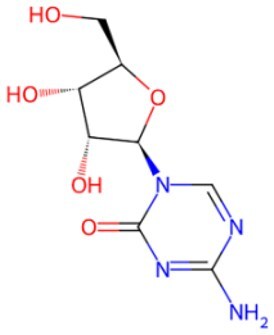	DNA methyltransferase (DNMT) inhibitor	Eliminating DNA methylation markers and activating pluripotency genes	Auxiliary	CSID: 9072	^26^
**Valproic Acid (VPA)**	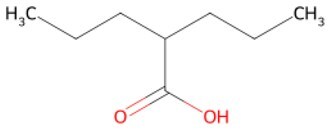	Histone deacetylase (HDAC) inhibitors	Enhance histone acetylation levels and promote chromatin opening	Driving/auxiliary	CSID: 3009	^27^
**Trichostatin A (TSA)**	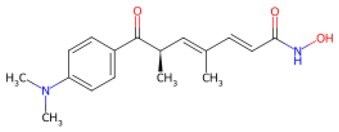	HDAC1/2-specific inhibitors	Synergistic enhancement of *Oct4* expression	Auxiliary	CSID: 392575	^28^
**5-Aza-2ʹ-deoxycitidine (5-aza-dc) or decitabine (DAC)**	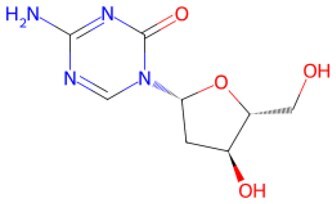	DNMT inhibitor	Activating pluripotency genes like *Oct4*	Auxiliary	CSID: 397844	^28^
**3-Deazaneplanocin (DZNep)**	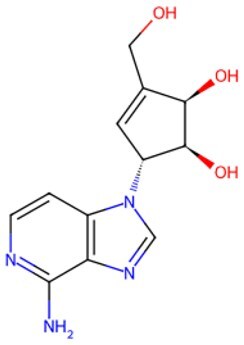	EZH2 inhibitor	Reducing H3K27me3 levels	Auxiliary	CSID: 65874	^29^ ^,^ ^30^
**Sodium butyrate**	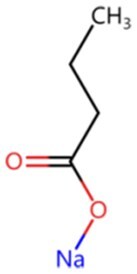	HDAC inhibitors	Synergistic with VPA to enhance chromatin plasticity	Auxiliary	CSID: 8727	^27^
**EPZ004777**	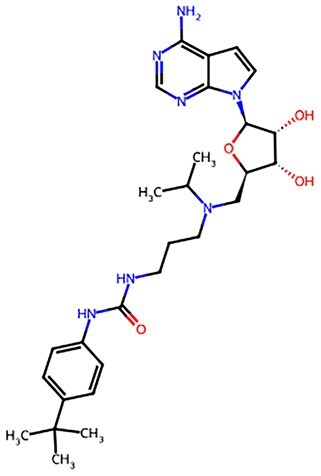	Selective DOT1L methyltransferase inhibitor	Inhibiting H3K79 methylation	Auxiliary	CSID: 28643044	^31^
**RG108**	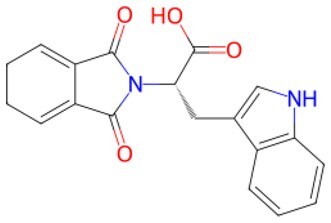	DNMT inhibitors	Reducing genome-wide methylation levels and activating silent pluripotency genes (e.g., *Oct4* and *Cdx2*)	Auxiliary	CSID: 17238013	^32^
**Tranylcypromine (TCP)**	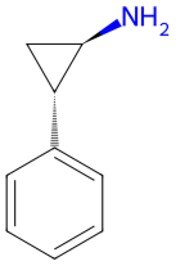	lysine-specific demethylase 1 (LSD1) inhibitor	Blocking H3K4me2 demethylation activates pluripotency genes	Driving	CSID: 18369	^33^
**SP2509**	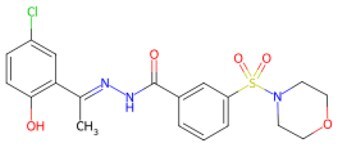	LSD1 inhibitor	Enhanced reprogramming	Driving/auxiliary	CSID: 31125416	^34^
**GSK126**	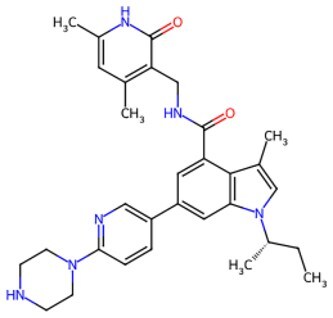	Inhibitors of H3K27me3 methylase (EZH2)	Reducing H3K27me3 levels	Auxiliary	CSID: 29763394	^35^
**IOX1**	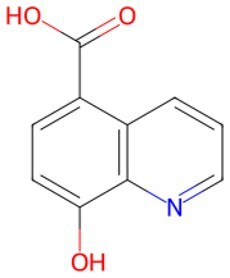	ALKBH5 inhibitor	Suppressing m6A RNA methylation and regulating metabolic reprogramming	Auxiliary	CSID: 404518	^36^
**BIX-01294**	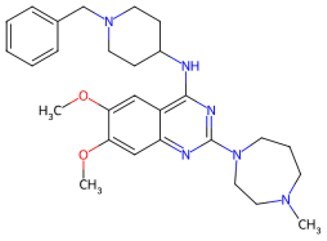	G9a histone methyltransferase inhibitor	Reducing H3K9me2 modification and promoting cell reprogramming	Auxiliary/driving	CSID: 24634766	^37^
**Tazemetostat (EPZ-6438)**	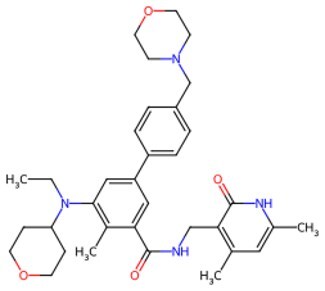	EZH2 inhibitor	Decreasing H3K27me3 levels and lifting differentiation gene inhibition	Auxiliary	CSID: 30208713	^38^
**JIB-04**	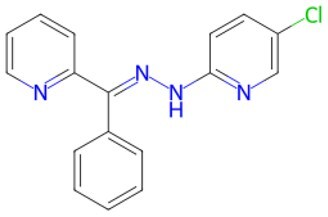	Jumonji histone demethylase broad-spectrum inhibitor	Increasing H3K9me3 levels and inhibiting differentiation	Auxiliary	CSID: 5011685	^39^
**AS-8351**	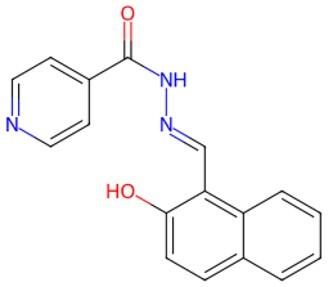	KDM5B inhibitor	Elevating H3K4me3 levels and regulating metabolic reprogramming	Auxiliary	CSID: 10943372	^40^
**A366**	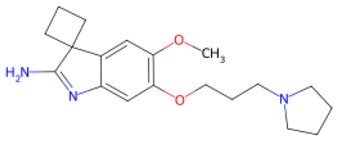	Histone lysine methyltransferase 2 (Ehmt2, also known as G9A) inhibitor	Partially conquering the reprogramming defects by altering the epigenetic and transcriptomic states	Auxiliary	CSID: 31131786	^41^
**JQ1**	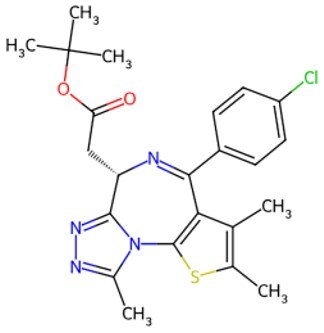	BET inhibitors	Increasing reprogramming	Auxiliary	CSID: 26323622	^42^
**Signaling pathway modulators**						
**CHIR99021**	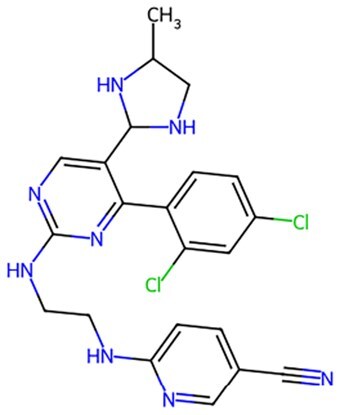	GSK-3β inhibitor activates Wnt/β-catenin pathway	Maintaining stem cell pluripotency	Driving	CSID: 84393685	^43^
**RepSox (E-616452)**	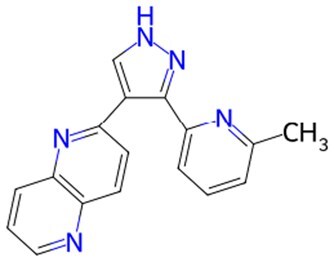	TGF-β receptor inhibitor	Blocking Smad2/3 phosphorylation and replacing Sox2	Driving	CSID: 395681	^44^
**SB431542**	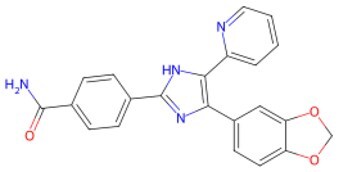	TGF-β receptor inhibitor	Blocking Smad2/3 signaling and inhibiting differentiation	Driving	CSID: 3716512	^45^
**A83-01**	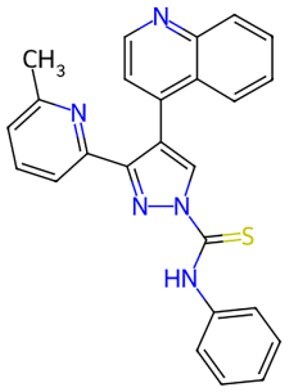	TGF-β receptor inhibitor	Blocking ALK4/5/7 activity and inhibiting differentiation	Driving	CSID: 17346282	^45^
**Dorsomorphin**	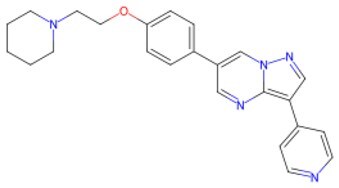	BMP inhibitor	Regulates mesodermal differentiation and promoting cell reprogramming	Auxiliary	CSID: 9698930	^46^ ^,^ ^47^
**PD0325901**	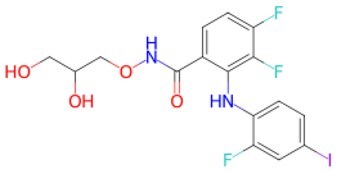	MEK inhibitor	Maintaining cellular pluripotency	Auxiliary	CSID: 10814340	^48^
**Forskolin**	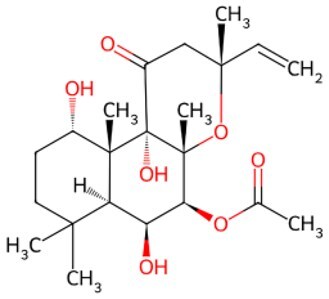	cAMP activator	Elevating cAMP levels, activating the PKA/CREB pathway, promoting proliferation, and synergistically/substituting for some functions of Klf/c-Myc	Driving	CSID: 43607	^49^
**SP600125**	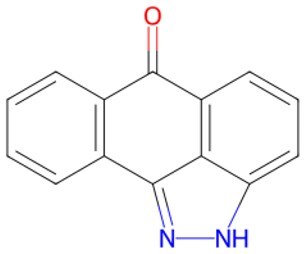	JNK inhibitor	Unraveling transcriptional repression of pluripotency genes to elevate reprogramming efficiency	Auxiliary	CSID: 8201	^50^
**LDN-193189**	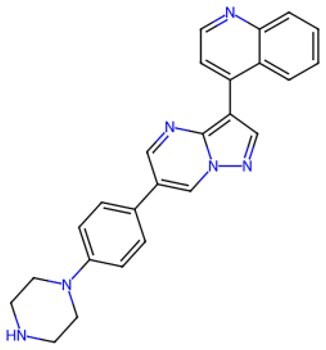	BMP inhibitor	Promoting neural differentiation and inhibiting mesenchymal lineage differentiation	Auxiliary	CSID: 24715454	^51^
**LY294002**	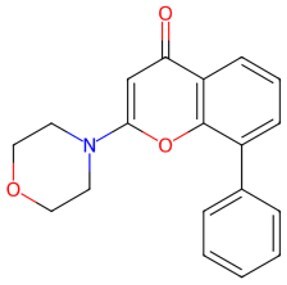	PI3K inhibitor	Boosting somatic cell reprogramming efficiency	Auxiliary	CSID: 3835	^52^
**PP242**	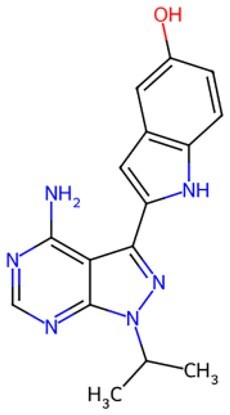	mTOR inhibitor	Facilitating somatic cell reprogramming	Auxiliary	CSID: 21437059	^52,53^
**Rapamycin**	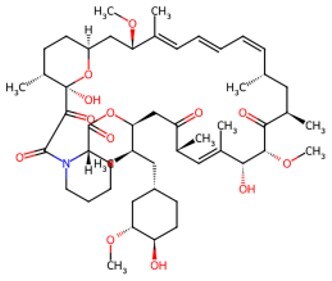	mTOR inhibitor	Promoting somatic cell reprogramming	Auxiliary	CSID: 58145102	^52,53^
**Metabolic modulators**						
**Vitamin C (Ascorbic Acid)**	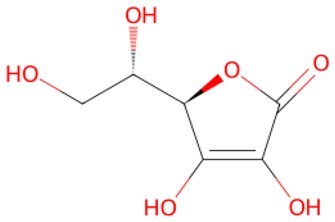	A dioxygenase-driven cofactor, participating in collagen prolyl hydroxylases, HIF prolyl hydroxylases, and histone demethylases	Promoting iPSC generation	Auxiliary	CSID: 10189562	^54^
**5-Aminoimidazole-4-carboxamide ribofuranoside (AICAR)**	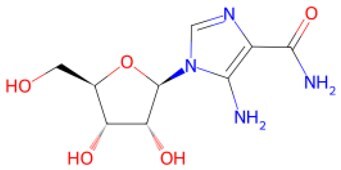	AMPK activator	Provoking differentiation of skeletal muscle progenitors	Auxiliary	CSID: 16560	^55^
**NR**	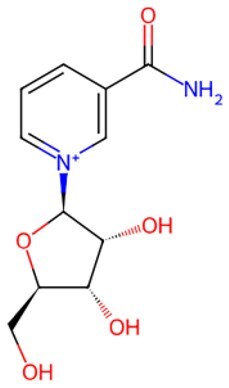	Induction of mitochondrial unfolded protein response and prohibition of protein synthesis	Promoting dysfunctional stem cells reprogramming	Auxiliary	CSID: 388956	^56^
**α-Ketoglutarate**	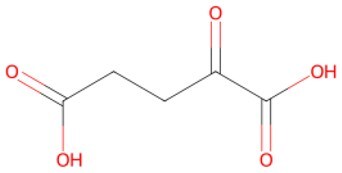	A key metabolic cofactor for tricyclic acid (TCA) cycle metabolites	Facilitating hair cell reprogramming	Auxiliary	CSID: 50	^57^
**Cell cycle and apoptosis regulators**						
**ABT-263 (Navitoclax)**	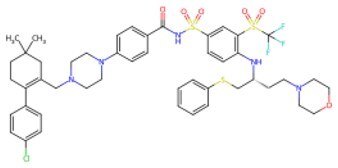	BCL-2 inhibitor	Selectively removing inflammatory and senescent cells to optimally reprogram the micro-environment	Auxiliary	CSID: 21864722	^58^
**Thiazovivin**	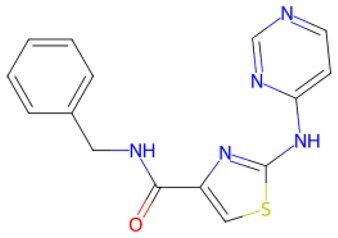	A Rho Kinase (ROCK) inhibitor	Improving cell reprogramming efficiency	Auxiliary	CSID: 24597612	^59^
**Y-27632**	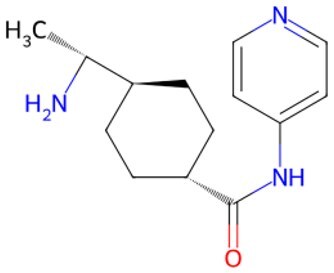	ROCK inhibitor	Boosting reprogrammed cell survival	Auxiliary	CSID: 20016532	^60^
**Kenpaullone**	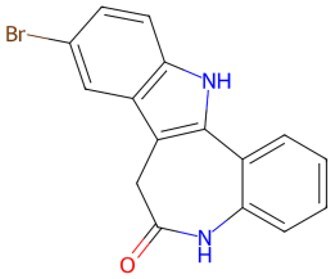	CDK/GSK-3β inhibitor	Increasing reprogramming efficiency of iPSCs	Auxiliary/potential driving	CSID: 3688	^61^
**AZD-5438**	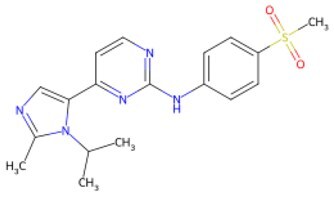	CDK2 inhibitor	Elevating high blastocyst formation rate in SCNT experiment	Auxiliary	CSID: 20578901	^62^
**Others**						
**BayK8644**	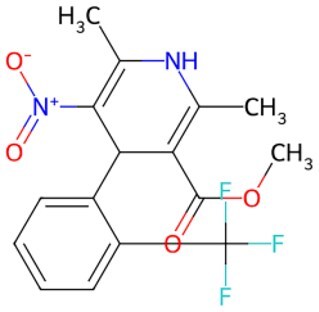	A L-channel calcium agonist	Improving reprogramming efficiency	Auxiliary	CSID: 2213	^63^
**Retinoic Acid (RA)**	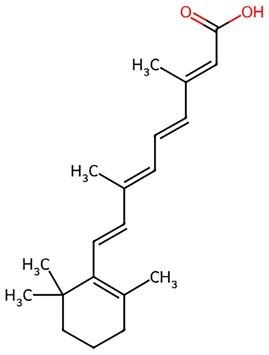	Activation of retinoic acid receptors (RAR)	Profoundly promoting reprogramming in MEFs	Driving	CSID: 392618	^64^
**BAY 11-7082**	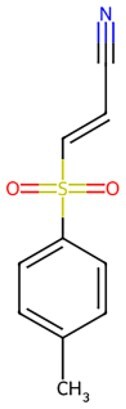	Strengthening OCT4 synthetic mRNA expression	Maintaining pluripotency and differentiation	Auxiliary	CSID: 4510086	^65^

### Telomerase reverse transcriptase activators

It has been suggested that the ability of stem cells to sustain tissue regeneration is directly relevant to telomere length and telomerase activity, playing a responsible role in supporting organ homeostasis and survival. Reintroduction of telomerase into the mice promotes stem cell regeneration and extends normal lifespan.^66^ A small-molecule, TERT-activating compound, TAC, reduces senescence-associated markers and promotes DNMT3B-mediated promoter hypermethylation to silence p16INK4a expression via reprogramming gene levels.^67^

### Epigenetic remodeling for anti-aging

#### Histone deacetylases inhibitors

Small molecules such as valproic acid (VPA) and TSA reset the epigenetic state of cells by inhibiting histone deacetylases (HDACs), restoring the gene expression of young cells.^68^ VPA combined with other small molecules not only restores the proliferative capacity of somatic cells though HDACs inhibition, chromatin openness remodeling, and the activation of pluripotency genes (e.g., *Oct4*, *Nanog*) but also breaks cellular aging barrier required for the induction of pluripotency, thereby promoting cell proliferation and inhibiting apoptosis.^69^^,^^70^ Although TSA does not directly act on class III HDACs (e.g., SIRT1, SIRT2), the overall elevation of histone acetylation levels may indirectly affect SIRT activity or downstream pathways through epigenetic reprogramming. For example, acetylation of NLRP3 is associated with aging-related chronic inflammation, and TSA may reverse aging-related inflammation as well as insulin resistance by upregulating SIRT2 and inhibiting NLRP3 activation.^71^^,^^72^

#### Methyltransferase inhibitors

Research has shown that GSK126, an EZH2 methyltransferase inhibitor, restores the transcriptional activity of the silenced gene by targeting EZH2, significantly reducing the aberrant H3K27me3 levels.^35^ GSK126 supplementation enhances β-cell maturation and provides regenerative β-cell replacing depleted or senescent β-cells in both juvenile and adult patients with type 1 diabetes.^38^ Moreover, vitamin C promotes DNA demethylation by enhancing TET enzyme activity, thereby optimizing the reprogramming process and indirectly influencing aging-related epigenetic modifications.^73^ Research indicates that vitamin C promotes pluripotency by regulating epigenetic states, potentially serving to reverse age-related cellular conditions.^74^ Importantly, in human skin models, vitamin C promotes DNA demethylation of genes associated with cell proliferation by maintaining TET enzyme activity, thereby thickening the epidermis and counteracting age-related skin thinning.^75^

#### Targeting miRNAs

Interestingly, miRNAs play critical roles in constructing pluripotency networks and reprogramming somatic cells into pluripotency.^76^^,^^77^ Transfection with mature miRNAs (miR-200c, miR-302s, and miR-369s) enables to successfully generate iPSCs.^78^ Notably, certain small molecules manipulate gene expression to influence cell fate by regulating miRNA production or degradation, thereby altering their cellular levels.^79^ For example, MEK inhibitor PD0325901 influences ESC self-renewal and cellular differentiation by regulating multiple miRNAs.^48^

Therefore, small-molecule compounds allow for the changeover of the “epigenetic age” of cells and the restoration of cellular function by the modulation of epigenetic modifications.

### Metabolic network remodeling for anti-aging

Metabolic remodeling is the foundation of cellular reprogramming and one of the central characteristics of senescence. During aging, the cellular metabolic network experiences remarkable reconstruction, which is mainly manifested by decreased mitochondrial oxidative phosphorylation efficiency, reduction of the NAD+/NADH ratio, altered signal pathways, and disorders of glucose, lipid, and amino acid metabolism.^80–84^ Metabolic network remodeling creates a positive feedback loop with epigenetic alterations and senescent cells, collectively driving tissue functional decline. Small molecules have demonstrated the potential to delay aging and extend healthy lifespan by targeting the metabolic remodeling network.

#### NAD+ precursor compounds

Study indicated that NAD+ repletion enhances and rejuvenates muscle stem cells function by reprogramming dysfunctional muscle stem cells, extending mammalian lifespan.^56^ Additionally, restoring mitochondrial NAD+ levels fights stem cell aging and promotes reprogramming of senescent somatic cells.^85^ Long-term NMN (a pivotal NAD+ intermediate) supplementation changes age-related gene expression and obviously ameliorates eye function and other pathological changes in aged mice via regulating energy metabolism–associated metabolic remodeling way.^86^ Moreover, supplementing with NMN regulates lipid metabolism, inflammation, fibrosis, cardiac dysfunction, and systemic insulin resistance, thereby indirectly promoting anti-aging effects.^87^^,^^88^ Another key NAD+ precursor, NR administration also restores metabolic and senescence-associated disorders by increasing in NAD+ levels and activation of SIRT1/3.^89^ The role of NAD+ metabolism in regulating mammalian cell aging has been clearly exhibited by Jun Yoshino’s team.^90^

### Regulator of signaling pathways

The process of embryonic development covers the selective expression of numerous genes and the switching of multiple signaling pathways, while reprogramming proceeds in the opposite direction and shares some co-regulatory pathways with the controllers of biological lifespan. Small-molecule compounds can manipulate cell fate by targeting critical links in following aspects. (1) Wnt signaling pathway: GSK-3β inhibitor participates in the regulation of the Wnt signaling pathway and is a key molecule for mouse somatic cells transformation to iPSCs.^9^ Notably, GSK-3β is upregulated and actuated in old mice, and its depletion mitigates senescence and glomerular aging, suggesting that GSK-3β inhibitors extend glomerular function by improving aging glomeruli.^91^ (2) TGF-β signaling pathway: This pathway exhibits bidirectional regulation in cell reprogramming. In classical reprogramming of somatic cells to iPSCs, TGF-β signaling inhibitor hinders the expression of differentiation-related genes and advances the activation of pluripotency networks by TFs.^44^^,^^92^ In contrast, in lineage-specific reprogramming (e.g., fibroblast-to-cardiomyocyte transformation), TGF-β enhances cellular plasticity through activation of EMT and facilitates trans-germinal fate switching.^93^ Additionally, a recent study proposes a model for the stepwise regulation of TGF-β in the naïve-primed pluripotency-differentiation of stem cells.^94^ Therefore, the therapeutic effect of TGF-β agonists or inhibitors on aging depends on the cellular state and integration of signaling pathways. However, upregulation of TGF-β leads to fibrosis, tissue decline, inflammatory response, and cellular senescence.^95^ (3) JNK signaling pathway: As a member of the MAPK family, JNK participates in early cellular reprogramming stages by phosphorylating TFs and regulating epigenetic modifying enzymes. It is considered to be a major obstacle to chemical reprogramming, as its suppression is essential for the induction of cellular plasticity and similar regenerative programs through the inhibition of pro-inflammatory pathways.^70^ During cell reprogramming, the dynamic regulation of JNK signaling pathway activity critically determines the efficiency of senescence-related molecular barrier breaking. Studies demonstrate that over-activation of the JNK pathway creates reprogramming resistance by phosphorylating the transcription factor c-Jun and upregulating senescence-associated genes such as p53/p21.^70^^,^^96^ Selectively using JNK inhibitor SP600125, inflammatory responses and immunoreactivity of NLRP3 can be reduced, while enhancing the survival efficiency of cellular reprogramming, allowing senescent cells to regain their proliferative potential.^97^^,^^98^ (4) mTOR signaling pathway: early stages of reprogramming require a metabolic shift from slow but efficient mitochondrial oxidative phosphorylation (OXPHOS) to inefficient but rapid glycolysis unique to pluripotent stem cells.^99^ Inhibition of the mTOR signaling pathway, one of the central pathways regulating cell growth, metabolism, and fate, improves the efficiency of iPSC reprogramming and longevity.^52^ mTORC1 suppression defer aging by triggering autophagy, which removes senescent and damaged mitochondria (mitophagy), and mitophagy accumulation correlates with senescence and related pathologies.^100^ Moreover, mTORC1 inhibitor (e.g., rapamycin) effectively boost intracellular antioxidant defenses, preventing skin aging by restraining the senescence phenotype SA-β-gal, reducing P53 and MMP-1 protein levels, while enhancing SOD and HO-1 activities.^101^

Molecular docking was employed to further evaluate the affinity of GSK-3β inhibitor, TGF-β inhibitor, mTOR inhibitor, and JNK inhibitor for p16INK4a. Supplementary figure 1 illustrates the binding positions of four small molecules to the amino acid residues of p16INK4a.

### SASP remodeling for anti-aging

SASP is a collection of inflammatory factors secreted by senescent cells that drive chronic inflammation and tissue degeneration. SASP has a dual role in the aging microenvironment: pro-aging and pro-regenerative. On one hand, chronic inflammation accelerates tissue fibrosis and stem cell dysfunction, while on the other hand, SASP factors promote tissue repair during regenerative processes.^102^ For example, IL-6 supports hepatocyte proliferation by activating STAT3 signaling.^103^ Recent study has revealed that small-molecule compounds such as JNK inhibitor SP600125 not only directly induce cellular reprogramming but also improve the senescence microenvironment or promote regeneration by modulating IL-6 and IL1β.^104^ Moreover, SP600125 significantly reduced active JNK levels and further prevented the influence of IL-17A on FTO expression to inhibit endothelial cell senescence.^105^

In addition to JNK inhibitor, a specific NF-кB inhibitor BAY 11-7082 decreases MMPs levels to treat senescent hepatocytes.^106^ Another study reports that BAY 11-7082 inhibits IL6 secretion by senile microglia, thereby alleviating senescence in HMC3 cells.^107^ The BET inhibitor JQ1 also blocks NF-кB-driven SASP by inhibiting BET protein binding to acetylated histones.^108^ Systemic supplementation with JQ1 notably reduced inflammatory cytokine levels, which is crucial for preventing macrophage senescence and removing LPS-induced lipid accumulation in senescent macrophages.^109^ These findings suggest that compounds that induce cellular reprogramming can modulate SASP, thereby attenuating cellular senescence progression.

### Cell cycle protein remodeling for anti-aging

Cell cycle proteins (Cyclins) and their dependent kinases (CDKs) exert an essential role in cell reprogramming (regaining pluripotency) and senescence (proliferative arrest) by precisely regulating cell cycle progression.^110^ Cell cycle arrest is a hallmark feature of senescent cells, manifested by G1/S phase block (p16/p21 upregulation) and loss of proliferative capacity. However, reprogramming requires transient release of cycle arrest to activate pluripotency genes and subsequent restoration of proliferative capacity. Thus, promotion of the G1/S phase transition and restoration of cell cycle progression are indispensable for reprogramming and reversal of aging.

Small-molecule compounds can both promote reprogramming of senescent cells to a functionally rejuvenated state by dynamically modulating cell cycle proteins and remove senescent cells. As a GSK3β inhibitor, CHIR99021 stabilizes β-catenin, enhances its nuclear translocation, and activates Wnt target genes (e.g. Cyclin D1) to drive the G1/S phase transition. Inhibition of GSK3β reduces phosphorylated degradation of β-catenin and prolongs pro-proliferative signaling transduction.^111^ In addition, as a cofactor for TET enzymes, vitamin C promotes DNA demethylation while inhibiting the p53-p21 pathway and lifting the G1 phase block. Vitamin C also reduces ROS and protects cells from oxidative damage associated with cycle block.^54^ Moreover, in human embryonic stem cell culture, ROCK inhibitor Y-27632 significantly decreases cell apoptosis and elevates single-cell survival rate, supporting clone expansion during reprogramming.^112^ Y-27632 upregulates cyclin D1, CDK4, and cyclin E to accelerate cell cycle progression transition, by activating extracellular signal-mediating kinase signaling cascades, thus inducing astrocyte proliferation.^113^

**Figure 2.**
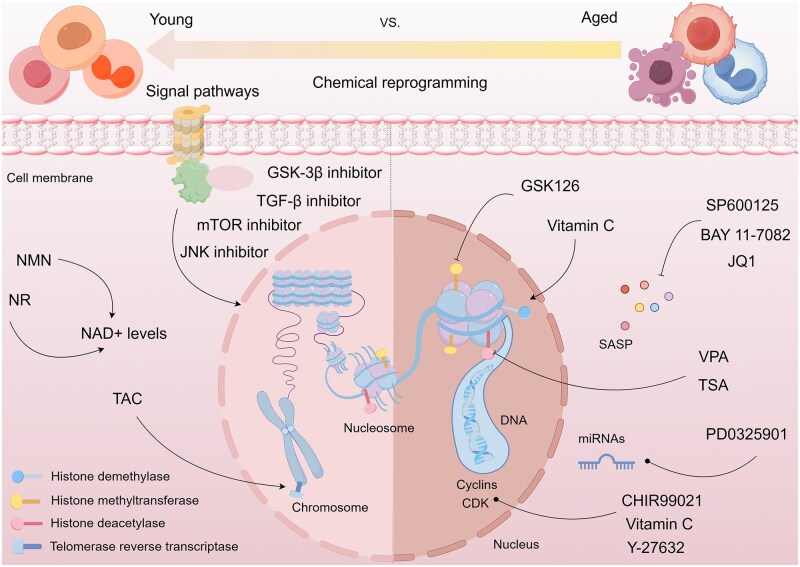
Mechanisms of action of representative small-molecule compounds for cell reprogramming and synchronized anti-aging. GSK126 (CSID: 29763394), JQ1 (CSID: 26323622), CHIR99021 (CSID: 84393685), VPA (CSID: 3009), TSA (CSID: 392575), PD0325901 (CSID: 10814340), SP600125 (CSID: 8201), Vitamin C (CSID: 10189562), NR (CSID: 388956), Y-27632 (CSID: 20016532), BAY 11-7082 (CSID: 4510086), and NMN (CSID: 13553).

Hence, small-molecule compounds exhibit anti-aging potential in reversing cell cycle arrest and inducing functional reprogramming by targeting cell cycle proteins. Figure 2 summarizes the mechanism of action of certain small-molecule compounds that reprogram cell fate against aging.

## Preclinical and clinical trials

Recent research indicates that specific combinations of small-molecule compounds induce various cell types to differentiate into other functional cells, which can be utilized in anti-aging, regenerative medicine, and treatment of other diseases. Table 2 summarizes the latest animal study data from 2023 to 2025, particularly regarding the in vivo reprogramming efficiency, tissue specificity, and safety of small molecules.

**Table 2.** The animal experiments from 2023–2026 about chemical reprogramming for anti-aging, regenerative medicine and other diseases.

The administered species (in vivo)	Starting cell types	Obtained cell types	Small molecules	Efficiency of reprogramming	Oncogenic risk	References
**Mice with radiation-induced salivary gland (SG) hypofunction**	Human SG cells	SG epithelial basal progenitor cells	Y-27632, A83-01, and LDN193189	No specific data was reported	Not reported, but the treatment successfully improved SG function	^114^
**A CB-17 wild-type mouse model of acute ischemic stroke**	Human monocyte-derived macrophages	Neuronal cells	CHIR99021, Dorsomorphin, Forskolin, isoxazole-9 (ISX-9), Y27632, and DB2313	Low (approximately 30%)	Not reported, but the therapy significantly improved neurological prognosis apart from weight loss and low appetite	^115^
**BALB/c nude mice with full-thickness burn injury**	Human epidermal keratinocytes	Sweat glands cells	CHIR99021, PMA, A83-01, forskolin, VPA, and TCP	With a > 70% production within 14 days.	Not reported, but it effectively promoted the regeneration of functional sweat gland cells	^116^
**Adult or aged mice with crush injury**	Mouse’s astrocytes	Neuronal cells	LDN193189 and CHIR99021	Low, data not shown	Not reported, but just two compounds successfully converted reactive astrocytes into neurons in the damaged spinal cord of adult mice	^117^
**The adult 129SvJ mice with left anterior descending coronary artery ligation**	Cardiomyocytes	Regenerative cardiac cells	CHIR99021 and A-485	No specific data was reported	Not reported, but the treatment in adult mouse hearts remarkably elevated survival and improved heart function post-myocardial infarction	^43^
**C57BL/6 mice with concanavalin A–induced liver failure or fah‐deficient mice**	Mouse embryonic fibroblasts	Functional hepatocyte‐like cells	CHIR99021, 616452, Forskolin, AM580, and EPZ004777 (C6FAE) or CHIR99021, 616452, Forskolin, CH55, UNC0638, and EPZ004777 (C6F5UE)	Low, 0.2%–0.8% of the initial mouse embryonic fibroblasts were converted to chemically induced hepatocytes	Not reported, but this approach demonstrated the potential of chemical reprogramming in organ/tissue repair and regeneration therapies.	^118^
**C57BL/6 mice with an HFD (60 % fat)**	Mouse embryonic fibroblasts	Adipocytes-like cells with brown/beige adipocyte properties	SB431542 (SB) and NKH477 (NK)	About 60%	Not reported, but this method alleviated the HFD-induced glucose metabolism disorders and insulin resistance, and promoted brown/beige adipocytes production	^119^

The clinical research on the induction of cell reprogramming by small-molecule compounds for anti-aging remains in the early exploratory stage. Although most findings focused on preclinical studies (animal models or cell experiments), some related single-molecule studies have entered clinical exploration. A phase 2 clinical trial (NCT04488601) demonstrated that long-term, low-dose intermittent rapamycin supplementation is relatively safe and significantly ameliorates lean tissue mass and pain in women.^120^ Another clinical trial (NCT03103893) revealed that rapamycin applied to the dorsal side of each hand every 24 to 48 h before bedtime effectively improves body tissue function and diminishes aging markers.^121^ These results support further studies in clinical settings where rapamycin supplementation may have similar advantages for older individuals.

Metabolic dysregulation represents a central hallmark of aging. In addition to inhibition of the overactive mTOR pathway, supplementation with NAD+ precursors restores energetic homeostasis and extends healthy lifespan. When NR is administered orally to older adults at 1000 mg per day for 6 weeks, it elevates NAD+ levels at the neuronal origin and decreases biomarkers associated with insulin resistance and neuroinflammatory pathways such as Aβ42, pJNK, and pERK1/2.^122^ Building upon NR’s established potential in alleviating Parkinson’s disease symptoms, an ongoing phase 2 trial (NCT06208527) examines its impact on frail elderly populations without neurodegenerative diseases through 2000 mg daily oral administration for one year. Positive findings regarding frailty improvement could carry substantial socio-health and economic implications, particularly given aging-related societal challenges. Similarly, compared to placebo, daily supplementing with 300, 600, or 900 mg of NMN increases the distance walked in a 6-minute walk test, decreases the biological age, and improves overall health.^123^

A recent Chinese clinical trial (NCT06794255) has garnered attention by investigating vitamin C—a significant small molecule for cell reprogramming induction—in anti-aging research. Participants take two 250 mg tablets of vitamin C or placebo daily after breakfast and dinner for 12 months, with face-to-face follow-ups every 6 months at designated locations. Telephone follow-ups occur bimonthly, including medication distribution, with participants required to disclose accurate medication adherence during each contact. These findings are highly anticipated. We have summarized the clinical trials of these small molecules used to induce cell reprogramming in the field of aging (Supplementary table 2) by visiting the https://clinicaltrials.gov/expert-search website, aiming to facilitate clinical translation and accelerate field development.

## Challenges and future directions

The core mechanism by which small-molecule compounds induce cellular reprogramming lies in their ability to mimic transcription factor functions, regulate intracellular signaling networks, and reverse aging-associated epigenetic alterations. Research indicates that specific combinations of small molecules can effectively activate pluripotency gene networks while simultaneously suppressing aging-related pathways, thereby achieving a reversal of cellular states.

First, small-molecule-compound-induced cellular reprogramming typically rewards the involvement of epigenetic modulators. Although the addition is not mandatory in all protocols—its necessity depends on factors such as reprogramming strategy, target cell type, and desired efficiency—epigenetic regulation plays a crucial role in cellular reprogramming. Research indicates that the reprogramming of fibroblasts often requires reversing differentiation-associated epigenetic barriers.^124^ Small-molecule epigenetic modulators actively clear these barriers: DNA methylation inhibitors (e.g., 5-aza-cytidine) reduce methylation levels at pluripotency gene promoters to enhance Oct4/Sox2 expression, while histone deacetylase (HDAC) inhibitors (e.g., VPA) increase histone acetylation, open chromatin structures, and accelerate reprogramming. Hongkui Deng et al. at Peking University combined a histone deacetylase inhibitor (VPA) with a histone demethylation inhibitor in their chemical induction protocol for mouse pluripotent stem cells, thereby crossing the epigenetic stability barriers in mouse somatic cells.^9^ Similarly, in the chemically induced human pluripotent stem cell protocol, the combined use of histone deacetylase inhibitor (VPA) and histone demethylating inhibitor has overcome the epigenetic stability limitations in human cells.^116^

Notably, epigenetic alterations have been identified as one of the core hallmarks of aging. During the aging process, the epigenome of cells and tissues undergoes significant and systematic changes. These alterations are not merely consequences of aging but also driving forces behind it. However, epigenetic modulators can reshape the epigenetic landscape of aging cells and reverse aging. Research has found that the fully chemical-free culture system of TCP (blocking H3K4me2 demethylation) and RepSox significantly reduces SA-β-gal activity in aged fibroblasts, upregulates pluripotency genes such as *OCT4* and *Nanog*, and simultaneously downregulates age-associated stress response genes *p21*, *p53*, and *IL6*. This epigenetic reprogramming not only restores cellular proliferative capacity but also improves oxidative stress and heterochromatin loss and reversing aging characteristics across multiple dimensions.^125^

**Figure 3.**
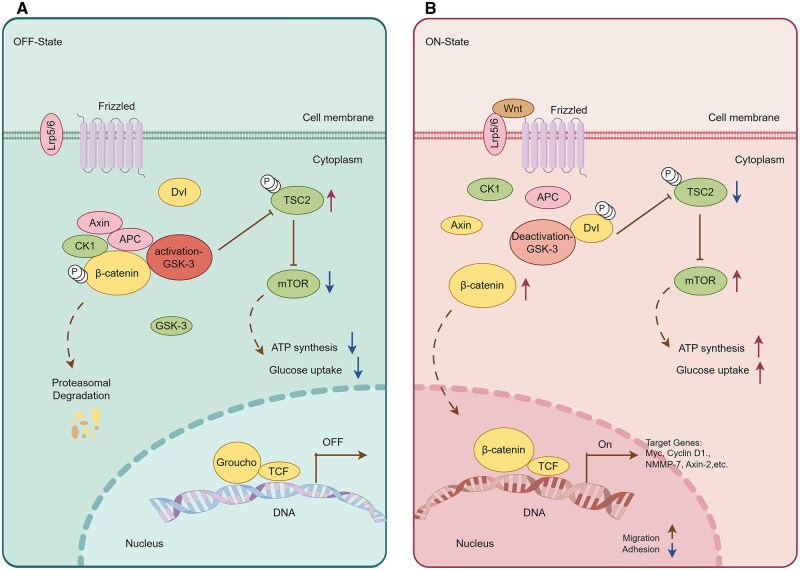
Regulation of growth and development by the Wnt-GSK3β-mTOR signaling pathway. (A) In the absence of Wnt signals, activated GSK3β not only phosphorylates β-catenin leading to its degradation but also phosphorylates and inhibits TSC2, which is a key inhibitor of mTORC1. (B) Upon binding to the Frizzled receptor and LRP5/6 co-receptor, the Wnt ligand inhibits the activity of the disrupt-in complex via the dishevelled protein. This leads to the accumulation of β-catenin in the cytoplasm and its subsequent translocation into the nucleus, where it initiates gene expression. Concurrently, Wnt signaling suppresses GSK3β, thereby releasing TSC2’s inhibition of mTORC1 and ultimately activating the mTORC1 complex.

Second, cellular signaling pathways serve as pivotal regulatory hubs in chemical reprogramming, precisely intervening in cellular fate by integrating epigenetic remodeling, metabolic reprogramming, and microenvironmental signals. Unlike the “hard switching” of genetic reprogramming (such as TFs), small molecules regulate signaling pathways more like a finely adjustable “dial,” enabling more precise and controllable spatiotemporal dynamic regulation. Among these, the Wnt signaling pathway stands out most prominently. Wnt signaling is an “on-off switch” for establishing and maintaining pluripotency. Activating the Wnt pathway during early reprogramming significantly enhances efficiency. In the canonical Wnt pathway, GSK-3β inhibitors (such as CHIR99021) prevent the formation of the β-catenin degradation complex (containing APC and Axin). Accumulated β-catenin enters the nucleus and binds to TCF/LEF TFs, activating pluripotency genes such as OCT4/SOX2/NANOG. This directly drives the conversion of somatic cells into iPSCs. Simultaneously, it activates Cyclin D1/c-Myc, accelerating the cell cycle process and shortening the reprogramming time. In addition, Wnt signaling regulates mitochondrial biogenesis via the mTOR pathway, providing energy support for reprogramming. Figure 3 displays the details of Wnt-GSK3β-mTOR signalling in cellular reprogramming and cell aging. It follows by the TGF-β signaling pathway, which is a key pathway for maintaining cellular differentiation and promoting EMT. RepSox (E-616452) and SB431542 are TGF-β receptor inhibitors that block Smad2/3 phosphorylation. This action releases suppression of pluripotency genes and promotes MET, a critical early event in cell reprogramming. Furthermore, inhibition of the TGF-β pathway synergistically interacts with Wnt pathway activation to clear obstacles for cellular identity conversion. Importantly, TGF-β activation can trigger aging. When hepatocytes age, factors like TGF-β diffuse throughout the body via the bloodstream, activating aging phenotypes in cells of other organs and inducing multi-organ aging (e.g., kidneys, brain).^126^ However, inhibiting TGF-β significantly reduces the expression of the aging marker p21 and improves the function of aging organs. Beyond the Wnt and TGF-β signaling pathways, JNK, BMP, and PI3K also exhibit crucial actions in cellular reprogramming. None of these signaling pathways operate independently. The success of chemical reprogramming in combating aging relies on constructing an ecosystem of interacting signaling pathways that simulates embryonic development.

The technology of small-molecule compounds inducing cell reprogramming has opened up a revolutionary pathway for anti-aging research. These small molecules can not only transform somatic cells to pluripotent stem cells in vitro but also partially alter the tissue aging phenotype in vivo. However, there are still multiple challenges from laboratory discoveries to clinical applications. Study shows global modification of chromatin by small-molecule compounds (e.g., VPA) may lead to non-target gene activation. For example, although the HDAC inhibitor VPA increases the efficiency of reprogramming, it simultaneously activates the proto-oncogene c-Myc and increases genomic instability.^127^ In addition, senescent cells are highly heterogeneous, with remarkably different epigenetic states and metabolic profiles among different cell types. Different combinations of molecules are needed to reverse multiple senescent cell types during reprogramming. For instance, reprogramming of human adult fibroblasts into neurons requires these seven small-molecule combinations including VPA, CHIR99021, RepSox, Forskolin, SP600125, GO6983, and Y-27632, whereas reprogramming of human astrocytes into neurons needs these four combinations of DAPT, CHIR99021, SB431542, and LDN193189.^128^^,^^129^ This limits the development of generalized therapeutic regimens. At the same time, it is not excluded that chemical reprogramming may induce long-term in vivo reprogramming, potentially triggering liver–intestinal failure and leading to premature death in model animals.^130^ To address these issues, researchers have developed nanocarrier-specific delivery systems, precise targeting strategies for heterogeneous senescent cells, and safer combinations of small molecules to enhance efficacy and reduce side effects. In recent years, various nanocarrier systems have been developed to enhance the efficiency of delivering small molecules to the brain, particularly for treating neurodegenerative diseases. It was found that acetylcholine-lipid nanoparticles (LNPs) exhibit excellent brain tropism. The mechanism involves synergistically enhancing intracellular mRNA delivery through functional binding to acetylcholine receptors (AchRs) and endocytosis.^131^ Similarly, stable polymeric siRNA nanomedicines modified with galactose (Gal-NP@siRNA) can enhance siRNA delivery across the blood–brain barrier by leveraging the circulation of glucose transporter 1 (Glut1) on cerebral vascular endothelial cells through transporter-mediated endocytosis, demonstrating high delivery efficiency and therapeutic efficacy.^132^ Given the high heterogeneity of senescent cells, senescent cells from different tissues and cell types express distinct surface markers. Researchers are encouraged to incorporate combinations of small molecules that recognize membrane proteins overexpressed on the surface of senescent cells, thereby enabling targeted intervention. Moreover, senescent cells typically alter their microenvironment through the SASP, promoting chronic inflammation and oxidative stress. It is possible to design prodrugs that activate only in highly reactive oxygen environments, or develop combinations capable of simultaneously delivering antioxidant drugs (such as NMN) and signaling pathway modulators. This approach repairs the microenvironment while initiating cellular reprogramming to combat aging, achieving synergistic effects.

For personalized and precision medicine, individual responses to small-molecule compounds may vary significantly due to differences in genetic backgrounds and environmental factors. Future research must explore personalized dosing regimens, tailoring reprogramming strategies based on an individual’s genetic background, epigenetic status, and metabolic characteristics. Organoid and organ-on-a-chip technologies provide platforms for personalized drug testing. Patient-derived cells allow the in vitro construction of organoid models to evaluate the reprogramming efficiency and safety of different small-molecule combinations.

Despite demonstrating immense potential in regenerative medicine and anti-aging research, chemical reprogramming technologies inevitably raise profound ethical concerns in clinical applications, necessitating rigorous evaluation and oversight throughout research and translation processes. Core ethical concerns primarily center on two aspects: the irreversibility of epigenetic reprogramming and the potential risks associated with germline editing. The core of chemical reprogramming is erasing and reconstructing epigenetic memory. While this fundamental reset of cellular identity represents a technical breakthrough, it carries ethical implications. Once a cell’s epigenetic clock is rewound and reprogrammed, its original identity and historical imprint are permanently erased. This raises philosophical questions about the integrity of an individual’s biological identity. Furthermore, the process may generate unpredictable off-target effects or epigenetic abnormalities, potentially leading to genomic imprinting disorders or tumorigenesis. These long-term risks remain difficult to fully assess and control at present. Applying chemical reprogramming to human gametes (sperm and eggs) or their precursor cells theoretically carries the risk of inadvertently modifying the germline (heritable genetic or epigenetic alterations). Such modifications could be passed on to future generations, causing permanent impacts on the human gene pool. Currently, the international community maintains a high level of vigilance regarding heritable germline editing. Chemical reprogramming technologies must strictly avoid any application scenarios that could lead to germline editing, unless conducted under extremely stringent regulatory oversight and with all safety and ethical concerns resolved. In response to these challenges, the international society for stem cell research (ISSCR) provided a crucial global regulatory framework in its updated 2021 guidelines for stem cell research and clinical translation. While these guidelines do not specifically address chemical reprogramming, their provisions concerning somatic cell reprogramming and embryonic research offer direct guidance.^133^

Although inducing cell reprogramming for anti-aging by small-molecule compounds face multiple challenges including efficacy, safety, and delivery, the future advancements in interdisciplinary innovations like artificial intelligence (such as deep learning models for predicting small-molecule-target interactions to accelerate personalized drug development), nanotechnology (such as engineered exosomes carrying anti-aging drugs), synthetic biology and multi-omics (such as combining DNA methylation clocks, plasma SASP factors, and metabolomics data to quantify anti-aging efficacy), and in accordance with ISSCR guidelines, the use of small-molecule cocktails for inducing cell reprogramming in anti-aging intervention treatments are expected to move from the laboratory to clinical practice.

## Supplementary Material

szaf069_Supplementary_Data

## References

[1] Li D , Shu X, Zhu P, et al. Chromatin accessibility dynamics during cell fate reprogramming. EMBO Rep. 2021;22:e51644. 10.15252/embr.202051644 33480184 PMC7857421

[2] Matoba S , Zhang Y. Somatic cell nuclear transfer reprogramming: mechanisms and applications. Cell Stem Cell. 2018;23:471–485. 10.1016/j.stem.2018.06.018 30033121 PMC6173619

[3] Briggs R , King TJ. Transplantation of living nuclei from blastula cells into enucleated frogs’ eggs. Proc Natl Acad Sci U S A. 1952;38:455–463. 10.1073/pnas.38.5.455 16589125 PMC1063586

[4] Gurdon JB. The developmental capacity of nuclei taken from intestinal epithelium cells of feeding tadpoles. J Embryol Exp Morphol. 1962;10:622–640. 13951335

[5] Weintraub H , Davis R, Tapscott S, et al. The myoD gene family: nodal point during specification of the muscle cell lineage. Science (New York, N.Y.). 1991;251:761–766. 10.1126/science.1846704 1846704

[6] Campbell KH , McWhir J, Ritchie WA, et al. Sheep cloned by nuclear transfer from a cultured cell line. Nature. 1996;380:64–66. 10.1038/380064a0 8598906

[7] Wilmut I , Schnieke AE, McWhir J, et al. Viable offspring derived from fetal and adult mammalian cells. Nature. 1997;385:810–813. 10.1038/385810a0 9039911

[8] Takahashi K , Yamanaka S. Induction of pluripotent stem cells from mouse embryonic and adult fibroblast cultures by defined factors. Cell. 2006;126:663–676. 10.1016/j.cell.2006.07.024 16904174

[9] Hou P , Li Y, Zhang X, et al. Pluripotent stem cells induced from mouse somatic cells by small-molecule compounds. Science (New York, N.Y.). 2013;341:651–654. 10.1126/science.1239278 23868920

[10] Warren L , Manos PD, Ahfeldt T, et al. Highly efficient reprogramming to pluripotency and directed differentiation of human cells with synthetic modified mRNA. Cell Stem Cell. 2010;7:618–630. 10.1016/j.stem.2010.08.012 20888316 PMC3656821

[11] Chakraborty S , Ji H, Kabadi AM, et al. A CRISPR/Cas9-based system for reprogramming cell lineage specification. Stem Cell Reports. 2014;3:940–947. 10.1016/j.stemcr.2014.09.013 25448066 PMC4264059

[12] Blackburn EH , Greider CW, Szostak JW. Telomeres and telomerase: the path from maize, tetrahymena and yeast to human cancer and aging. Nat Med. 2006;12:1133–1138. 10.1038/nm1006-1133 17024208

[13] Horvath S. DNA methylation age of human tissues and cell types. Genome Biol. 2013;14:R115. 10.1186/gb-2013-14-10-r115 24138928 PMC4015143

[14] Imai S , Guarente L. NAD+ and sirtuins in aging and disease. Trends Cell Biol. 2014;24:464–471. 10.1016/j.tcb.2014.04.002 24786309 PMC4112140

[15] Fulop T , Larbi A, Pawelec G, et al. Immunology of aging: the birth of inflammaging. Clin Rev Allergy Immunol. 2023;64:109–122. 10.1007/s12016-021-08899-6 34536213 PMC8449217

[16] Li Y , Tian X, Luo J, et al. Molecular mechanisms of aging and anti-aging strategies. Cell Commun Signal. 2024;22:285. 10.1186/s12964-024-01663-1 38790068 PMC11118732

[17] Melo Dos Santos LS , Trombetta-Lima M, Eggen B, et al. Cellular senescence in brain aging and neurodegeneration. Ageing Res Rev. 2024;93:102141. 10.1016/j.arr.2023.102141 38030088

[18] McHugh D , Gil J. Senescence and aging: causes, consequences, and therapeutic avenues. J Cell Biol. 2018;217:65–77. 10.1083/jcb.201708092 29114066 PMC5748990

[19] López-Otín C , Blasco MA, Partridge L, et al. Hallmarks of aging: an expanding universe. Cell. 2023;186:243–278. 10.1016/j.cell.2022.11.001 36599349

[20] Ocampo A , Reddy P, Martinez-Redondo P, et al. In vivo amelioration of age-associated hallmarks by partial reprogramming. Cell. 2016;167:1719–1733.e12. 10.1016/j.cell.2016.11.052 27984723 PMC5679279

[21] Menendez JA , Alarcón T. Senescence-inflammatory regulation of reparative cellular reprogramming in aging and cancer. Front Cell Dev Biol. 2017;5:49. 10.3389/fcell.2017.00049 28529938 PMC5418360

[22] Kane AE , Sinclair DA. Epigenetic changes during aging and their reprogramming potential. Crit Rev Biochem Mol Biol. 2019;54:61–83. 10.1080/10409238.2019.1570075 30822165 PMC6424622

[23] Zhao Z , Pan X, Liu L, et al. Telomere length maintenance, shortening, and lengthening. J Cell Physiol. 2014;229:1323–1329. 10.1002/jcp.24537 24374808

[24] Wang L , Xu X, Jiang C, et al. mTORC1-PGC1 axis regulates mitochondrial remodeling during reprogramming. Febs J. 2020;287:108–121. 10.1111/febs.15024 31361392

[25] Lee HJ , Gutierrez-Garcia R, Vilchez D. Embryonic stem cells: a novel paradigm to study proteostasis? Febs J. 2017;284:391–398. 10.1111/febs.13810 27398614

[26] De Carvalho DD , You JS, Jones PA. DNA methylation and cellular reprogramming. Trends Cell Biol. 2010;20:609–617. 10.1016/j.tcb.2010.08.003 20810283 PMC2981432

[27] Huangfu D , Maehr R, Guo W, et al. Induction of pluripotent stem cells by defined factors is greatly improved by small-molecule compounds. Nat Biotechnol. 2008;26:795–797. 10.1038/nbt1418 18568017 PMC6334647

[28] Hattori N , Nishino K, Ko YG, et al. Epigenetic control of mouse oct-4 gene expression in embryonic stem cells and trophoblast stem cells. J Biol Chem. 2004;279:17063–17069. 10.1074/jbc.M309002200 14761969

[29] Fiskus W , Wang Y, Sreekumar A, et al. Combined epigenetic therapy with the histone methyltransferase EZH2 inhibitor 3-deazaneplanocin a and the histone deacetylase inhibitor panobinostat against human AML cells. Blood. 2009;114:2733–2743. 10.1182/blood-2009-03-213496 19638619 PMC2756128

[30] Jing H , Liao L, An Y, et al. Suppression of EZH2 prevents the shift of osteoporotic MSC fate to adipocyte and enhances bone formation during osteoporosis. Mol Ther. 2016;24:217–229. 10.1038/mt.2015.152 26307668 PMC4817806

[31] Tao J , Zhang Y, Zuo X, et al. DOT1L inhibitor improves early development of porcine somatic cell nuclear transfer embryos. PLoS One. 2017;12:e0179436. 10.1371/journal.pone.0179436 28632762 PMC5478106

[32] Zarei M , Shamaghdari B, Vahabi Z, et al. Epigenetic reprogramming in cloned mouse embryos following treatment with DNA methyltransferase and histone deacetylase inhibitors. Syst Biol Reprod Med. 2022;68:227–238. 10.1080/19396368.2022.2036868 35382652

[33] Sun H , Liang L, Li Y, et al. Lysine-specific histone demethylase 1 inhibition promotes reprogramming by facilitating the expression of exogenous transcriptional factors and metabolic switch. Sci Rep. 2016;6:30903. 10.1038/srep30903 27481483 PMC4969595

[34] Yuan JB , Gu GX, Jin BM, et al. Menin maintains lysosomal and mitochondrial homeostasis through epigenetic mechanisms in lung cancer. Cell Death Dis. 2025;16:163. 10.1038/s41419-025-07489-0 40057469 PMC11890858

[35] Xie B , Zhang H, Wei R, et al. Histone H3 lysine 27 trimethylation acts as an epigenetic barrier in porcine nuclear reprogramming. Reproduction (Cambridge, England). 2016;151:9–16. 10.1530/rep-15-0338 26515777

[36] Cheng P , Han H, Chen F, et al. Amelioration of acute myocardial infarction injury through targeted ferritin nanocages loaded with an ALKBH5 inhibitor. Acta Biomater. 2022;140:481–491. 10.1016/j.actbio.2021.11.041 34879293

[37] Huang J , Zhang H, Yao J, et al. BIX-01294 increases pig cloning efficiency by improving epigenetic reprogramming of somatic cell nuclei. Reproduction (Cambridge, England). 2016;151:39–49. 10.1530/rep-15-0460 26604326

[38] Al-Hasani K , Marikar SN, Kaipananickal H, et al. EZH2 inhibitors promote β-like cell regeneration in young and adult type 1 diabetes donors. Sig Transduct Target Ther. 2024;9:2. 10.1038/s41392-023-01707-x PMC10757994 38161208

[39] Tran TA , Zhang QJ, Wang L, et al. Inhibition of Jumonji demethylases reprograms severe dilated cardiomyopathy and prolongs survival. J Biol Chem. 2022;298:101515. 10.1016/j.jbc.2021.101515 34933013 PMC8803621

[40] Zhang ZG , Zhang HS, Sun HL, et al. KDM5B promotes breast cancer cell proliferation and migration via AMPK-mediated lipid metabolism reprogramming. Exp Cell Res. 2019;379:182–190. 10.1016/j.yexcr.2019.04.006 30978340

[41] Wang J , Zhou C, Gao S, et al. Single-cell multiomics sequencing reveals the reprogramming defects in embryos generated by round spermatid injection. Sci Adv. 2022;8:eabm3976. 10.1126/sciadv.abm3976 35947654 PMC9365279

[42] Shao Z , Yao C, Khodadadi-Jamayran A, et al. Reprogramming by de-bookmarking the somatic transcriptional program through targeting of BET bromodomains. Cell Rep. 2016;16:3138–3145. 10.1016/j.celrep.2016.08.060 27653680 PMC13729475

[43] Zhou W , He K, Wang C, et al. Pharmacologically inducing regenerative cardiac cells by small molecule drugs. Elife. 2024;13: 10.7554/eLife.93405 PMC11627505 39651957

[44] Ichida JK , Blanchard J, Lam K, et al. A small-molecule inhibitor of Tgf-Beta signaling replaces sox2 in reprogramming by inducing nanog. Cell Stem Cell. 2009;5:491–503. 10.1016/j.stem.2009.09.012 19818703 PMC3335195

[45] Zeng J , Li Y, Ma Z, et al. Advances in small molecules in cellular reprogramming: effects, structures, and mechanisms. Curr Stem Cell Res Ther. 2021;16:115–132. 10.2174/1574888x15666200621172042 32564763

[46] Yu PB , Hong CC, Sachidanandan C, et al. Dorsomorphin inhibits BMP signals required for embryogenesis and iron metabolism. Nat Chem Biol. 2008;4:33–41. 10.1038/nchembio.2007.54 18026094 PMC2727650

[47] Chang D , Sun C, Tian X, et al. Regulation of cardiac fibroblasts reprogramming into cardiomyocyte-like cells with a cocktail of small molecule compounds. FEBS Open Bio. 2024;14:983–1000. 10.1002/2211-5463.13811 PMC11148126 38693086

[48] Ai Z , Shao J, Shi X, et al. Maintenance of self-renewal and pluripotency in J1 mouse embryonic stem cells through regulating transcription factor and MicroRNA expression induced by PD0325901. Stem Cells Int. 2016;2016:1792573. 10.1155/2016/1792573 26770202 PMC4685126

[49] Wang G , Zhang D, Qin L, et al. Forskolin-driven conversion of human somatic cells into induced neurons through regulation of the cAMP-CREB1-JNK signaling. Theranostics. 2024;14:1701–1719. 10.7150/thno.92700 38389831 PMC10879881

[50] Yao K , Ki MO, Chen H, et al. JNK1 and 2 play a negative role in reprogramming to pluripotent stem cells by suppressing Klf4 activity. Stem Cell Res. 2014;12:139–152. 10.1016/j.scr.2013.10.005 24211391

[51] Chambers SM , Qi Y, Mica Y, et al. Combined small-molecule inhibition accelerates developmental timing and converts human pluripotent stem cells into nociceptors. Nat Biotechnol. 2012;30:715–720. 10.1038/nbt.2249 22750882 PMC3516136

[52] Chen T , Shen L, Yu J, et al. Rapamycin and other longevity-promoting compounds enhance the generation of mouse induced pluripotent stem cells. Aging Cell. 2011;10:908–911. 10.1111/j.1474-9726.2011.00722.x 21615676

[53] Menendez JA , Vellon L, Oliveras-Ferraros C, et al. mTOR-regulated senescence and autophagy during reprogramming of somatic cells to pluripotency: a roadmap from energy metabolism to stem cell renewal and aging. Cell Cycle (Georgetown, Tex.). 2011;10:3658–3677. 10.4161/cc.10.21.18128 22052357

[54] Esteban MA , Wang T, Qin B, et al. Vitamin C enhances the generation of mouse and human induced pluripotent stem cells. Cell Stem Cell. 2010;6:71–79. 10.1016/j.stem.2009.12.001 20036631

[55] Belli R , Bonato A, De Angelis L, et al. Metabolic reprogramming promotes myogenesis during aging. Front Physiol. 2019;10:897. 10.3389/fphys.2019.00897 31354530 PMC6636331

[56] Zhang H , Ryu D, Wu Y, et al. NAD^+^ repletion improves mitochondrial and stem cell function and enhances life span in mice. Science (New York, N.Y.). 2016;352:1436–1443. 10.1126/science.aaf2693 27127236

[57] Liu Q , Zhang L, Chen Z, et al. Metabolic profiling of cochlear organoids identifies α-ketoglutarate and NAD(+) as limiting factors for hair cell reprogramming. Adv Sci (Weinheim, Baden-Wurttemberg, Germany). 2024;11:e2308032. 10.1002/advs.202308032 PMC11425867 38993037

[58] Liu Q , Wang F, Du Y, et al. p16INK4a deletion alleviated obesity-associated kidney fibrosis by regulating metabolic reprogramming and the inflammasome pathway. J Cell Mol Med. 2025;29:e70444. 10.1111/jcmm.70444 40079088 PMC11904428

[59] Mohseni R , Shoae-Hassani A, Verdi J. Reprogramming of endometrial adult stromal cells in the presence of a ROCK inhibitor, thiazovivin, could obtain more efficient iPSCs. Cell Biol Int. 2015;39:515–518. 10.1002/cbin.10411 25490878

[60] Wu X , Wang S, Li M, et al. Conditional reprogramming: next generation cell culture. Acta Pharm Sin B. 2020;10:1360–1381. 10.1016/j.apsb.2020.01.011 32963937 PMC7488362

[61] Pfisterer U , Ek F, Lang S, et al. Small molecules increase direct neural conversion of human fibroblasts. Sci Rep. 2016;6:38290. 10.1038/srep38290 27917895 PMC5137010

[62] Li XC , Guo Q, Zhu HY, et al. Parthenogenetic activation and somatic cell nuclear transfer of porcine oocytes activated by an electric pulse and AZD5438 treatment. Zygote (Cambridge, England). 2017;25:453–461. 10.1017/s0967199417000272 28712374

[63] Shi Y , Desponts C, Do JT, et al. Induction of pluripotent stem cells from mouse embryonic fibroblasts by Oct4 and Klf4 with small-molecule compounds. Cell Stem Cell. 2008;3:568–574. 10.1016/j.stem.2008.10.004 18983970

[64] Wang W , Yang J, Liu H, et al. Rapid and efficient reprogramming of somatic cells to induced pluripotent stem cells by retinoic acid receptor gamma and liver receptor homolog 1. Proc Natl Acad Sci U S A. 2011;108:18283–18288. 10.1073/pnas.1100893108 21990348 PMC3215025

[65] Awe JP , Crespo AV, Li Y, et al. BAY11 enhances OCT4 synthetic mRNA expression in adult human skin cells. Stem Cell Res Ther. 2013;4:15. 10.1186/scrt163 23388106 PMC3706837

[66] Siegl-Cachedenier I , Flores I, Klatt P, et al. Telomerase reverses epidermal hair follicle stem cell defects and loss of long-term survival associated with critically short telomeres. J Cell Biol. 2007;179:277–290. 10.1083/jcb.200704141 17954610 PMC2064764

[67] Shim HS , Iaconelli J, Shang X, et al. TERT activation targets DNA methylation and multiple aging hallmarks. Cell. 2024;187:4030–4042.e13. 10.1016/j.cell.2024.05.048 38908367 PMC11552617

[68] McIntyre RL , Daniels EG, Molenaars M, et al. From molecular promise to preclinical results: HDAC inhibitors in the race for healthy aging drugs. EMBO Mol Med. 2019;11:e9854. 10.15252/emmm.201809854 31368626 PMC6728603

[69] Zhai Y , Chen X, Yu D, et al. Histone deacetylase inhibitor valproic acid promotes the induction of pluripotency in mouse fibroblasts by suppressing reprogramming-induced senescence stress. Exp Cell Res. 2015;337:61–67. 10.1016/j.yexcr.2015.06.003 26112217

[70] Guan J , Wang G, Wang J, et al. Chemical reprogramming of human somatic cells to pluripotent stem cells. Nature. 2022;605:325–331. 10.1038/s41586-022-04593-5 35418683

[71] Kyrylenko S , Kyrylenko O, Suuronen T, et al. Differential regulation of the Sir2 histone deacetylase gene family by inhibitors of class I and II histone deacetylases. Cell Mol Life Sci. 2003;60:1990–1997. 10.1007/s00018-003-3090-z 14523559 PMC11138505

[72] He M , Chiang HH, Luo H, et al. An acetylation switch of the NLRP3 inflammasome regulates Aging-Associated chronic inflammation and insulin resistance. Cell Metab. 2020;31:580–591.e5. 10.1016/j.cmet.2020.01.009 32032542 PMC7104778

[73] Chen J , Guo L, Zhang L, et al. Vitamin C modulates TET1 function during somatic cell reprogramming. Nat Genet. 2013;45:1504–1509. 10.1038/ng.2807 24162740

[74] Blaschke K , Ebata KT, Karimi MM, et al. Vitamin C induces Tet-dependent DNA demethylation and a blastocyst-like state in ES cells. Nature. 2013;500:222–226. 10.1038/nature12362 23812591 PMC3893718

[75] Sato Y , Sato A, Kuwano A, et al. Vitamin C promotes epidermal proliferation by promoting DNA demethylation of proliferation-related genes in human epidermal equivalents. J Invest Dermatol. 2025;145:2775–2788.e14. 10.1016/j.jid.2025.03.040 40262671

[76] Luginbühl J , Sivaraman DM, Shin JW. The essentiality of non-coding RNAs in cell reprogramming. Noncoding RNA Res. 2017;2:74–82. 10.1016/j.ncrna.2017.04.002 30159423 PMC6096403

[77] Anokye-Danso F , Trivedi CM, Juhr D, et al. Highly efficient miRNA-mediated reprogramming of mouse and human somatic cells to pluripotency. Cell Stem Cell. 2011;8:376–388. 10.1016/j.stem.2011.03.001 21474102 PMC3090650

[78] Miyoshi N , Ishii H, Nagano H, et al. Reprogramming of mouse and human cells to pluripotency using mature microRNAs. Cell Stem Cell. 2011;8:633–638. 10.1016/j.stem.2011.05.001 21620789

[79] Li J , Zhang W, Zhou M, et al. Small molecules modulating biogenesis or processing of microRNAs with therapeutic potentials. Curr Med Chem. 2013;20:3604–3612. 10.2174/0929867311320290006 23745565

[80] Dato S , Hoxha E, Crocco P, et al. Amino acids and amino acid sensing: implication for aging and diseases. Biogerontology. 2019;20:17–31. 10.1007/s10522-018-9770-8 30255223

[81] Li Q , Xiao N, Zhang H, et al. Systemic aging and aging-related diseases. Faseb J. 2025;39:e70430. 10.1096/fj.202402479RRR 40022602

[82] Greco M , Villani G, Mazzucchelli F, et al. Marked aging-related decline in efficiency of oxidative phosphorylation in human skin fibroblasts. Faseb J. 2003;17:1706–1708. 10.1096/fj.02-1009fje 12958183

[83] Amjad S , Nisar S, Bhat AA, et al. Role of NAD(+) in regulating cellular and metabolic signaling pathways. Mol Metab. 2021;49:101195. 10.1016/j.molmet.2021.101195 33609766 PMC7973386

[84] Templeman NM , Murphy CT. Regulation of reproduction and longevity by nutrient-sensing pathways. J Cell Biol. 2018;217:93–106. 10.1083/jcb.201707168 29074705 PMC5748989

[85] Son MJ , Kwon Y, Son T, et al. Restoration of mitochondrial NAD(+) levels delays stem cell senescence and facilitates reprogramming of aged somatic cells. Stem Cells (Dayton, Ohio). 2016;34:2840–2851. 10.1002/stem.2460 27428041

[86] Mills KF , Yoshida S, Stein LR, et al. Long-term administration of nicotinamide mononucleotide mitigates age-associated physiological decline in mice. Cell Metab. 2016;24:795–806. 10.1016/j.cmet.2016.09.013 28068222 PMC5668137

[87] Tian X , Rong Y, Luo J, et al. Microbial creation of β-nicotinamide mononucleotide and its regulation of lipid metabolism in the liver of high-fat diet mice. Cell Biochem Funct. 2024;42:e4087. 10.1002/cbf.4087 38953407

[88] Wang S , Han Y, Liu R, et al. Glycolysis-mediated activation of v-ATPase by nicotinamide mononucleotide ameliorates lipid-induced cardiomyopathy by repressing the CD36-TLR4 axis. Circ Res. 2024;134:505–525. 10.1161/circresaha.123.322910 38422177 PMC10906217

[89] Cantó C , Houtkooper RH, Pirinen E, et al. The NAD(+) precursor nicotinamide riboside enhances oxidative metabolism and protects against high-fat diet-induced obesity. Cell Metab. 2012;15:838–847. 10.1016/j.cmet.2012.04.022 22682224 PMC3616313

[90] Yoshino J , Baur JA, Imai SI. NAD(+) intermediates: the biology and therapeutic potential of NMN and NR. Cell Metab. 2018;27:513–528. 10.1016/j.cmet.2017.11.002 29249689 PMC5842119

[91] Kreidberg JA , Schumacher VA. GSK3β and the aging kidney. J Clin Invest. 2022;132:10.1172/jci155885 PMC8843609 35166232

[92] Lin T , Ambasudhan R, Yuan X, et al. A chemical platform for improved induction of human iPSCs. Nat Methods. 2009;6:805–808. 10.1038/nmeth.1393 19838168 PMC3724527

[93] Werner JH , Rosenberg JH, Um JY, et al. Molecular discoveries and treatment strategies by direct reprogramming in cardiac regeneration. Transl Res. 2019;203:73–87. 10.1016/j.trsl.2018.07.012 30142308 PMC6289806

[94] Zhao B , Yu X, Shi J, et al. A stepwise mode of TGFβ-SMAD signaling and DNA methylation regulates naïve-to-primed pluripotency and differentiation. Nat Commun. 2024;15:10123. 10.1038/s41467-024-54433-5 39578449 PMC11584862

[95] Tominaga K , Suzuki HI. TGF-β signaling in cellular senescence and aging-related pathology. Int J Mol Sci. 2019;20:10.3390/ijms20205002 PMC6834140 31658594

[96] Dhanasekaran DN , Reddy EP. JNK signaling in apoptosis. Oncogene. 2008;27:6245–6251. 10.1038/onc.2008.301 18931691 PMC3063296

[97] He J , Liu T, Li Y, et al. JNK inhibition alleviates delayed neurocognitive recovery after surgery by limiting microglia pyroptosis. Int Immunopharmacol. 2021;99:107962. 10.1016/j.intimp.2021.107962 34298396

[98] Wan XY , Xu LY, Li B, et al. Chemical conversion of human lung fibroblasts into neuronal cells. Int J Mol Med. 2018;41:1463–1468. 10.3892/ijmm.2018.3375 29328434 PMC5819915

[99] Folmes CD , Nelson TJ, Martinez-Fernandez A, et al. Somatic oxidative bioenergetics transitions into pluripotency-dependent glycolysis to facilitate nuclear reprogramming. Cell Metab. 2011;14:264–271. 10.1016/j.cmet.2011.06.011 21803296 PMC3156138

[100] Saxton RA , Sabatini DM. mTOR signaling in growth, metabolism, and disease. Cell. 2017;168:960–976. 10.1016/j.cell.2017.02.004 28283069 PMC5394987

[101] Tang Y , Yang S, Qiu Z, et al. Rapamycin attenuates H(2)O(2)-induced oxidative stress-related senescence in human skin fibroblasts. Tissue Eng Regen Med. 2024;21:1049–1059. 10.1007/s13770-024-00660-2 39093548 PMC11416443

[102] Lopes-Paciencia S , Saint-Germain E, Rowell MC, et al. The senescence-associated secretory phenotype and its regulation. Cytokine. 2019;117:15–22. 10.1016/j.cyto.2019.01.013 30776684

[103] Cheng N , Kim KH, Lau LF. Senescent hepatic stellate cells promote liver regeneration through IL-6 and ligands of CXCR2. JCI Insight. 2022;7:10.1172/jci.insight.158207 PMC9431681 35708907

[104] Kawamura K , Matsumura Y, Kawamura T, et al. Endometrial senescence is mediated by interleukin 17 receptor B signaling. Cell Commun Signal. 2024;22:363. 10.1186/s12964-024-01740-5 39010112 PMC11247761

[105] Li N , Luo R, Zhang W, et al. IL-17A promotes endothelial cell senescence by up-regulating the expression of FTO through activating JNK signal pathway. Biogerontology. 2023;24:99–110. 10.1007/s10522-022-09999-2 36463389

[106] Zang J , Sha M, Zhang C, et al. Senescent hepatocyte secretion of matrix metalloproteinases is regulated by nuclear factor-κB signaling. Life Sci. 2017;191:205–210. 10.1016/j.lfs.2017.10.023 29054454

[107] Cook M , Lin H, Mishra SK, et al. Bay 11-7082 inhibits the secretion of interleukin-6 by senescent human microglia. Biochem Biophys Res Commun. 2022;617:30–35. 10.1016/j.bbrc.2022.05.090 35671608 PMC9540971

[108] Meng S , Zhang L, Tang Y, et al. BET inhibitor JQ1 blocks inflammation and bone destruction. J Dent Res. 2014;93:657–662. 10.1177/0022034514534261 24799421 PMC4107547

[109] Wang H , Fu H, Zhu R, et al. BRD4 contributes to LPS-induced macrophage senescence and promotes progression of atherosclerosis-associated lipid uptake. Aging (Albany NY). 2020;12:9240–9259. 10.18632/aging.103200 32392533 PMC7288959

[110] Jirawatnotai S , Dalton S, Wattanapanitch M. Role of cyclins and cyclin-dependent kinases in pluripotent stem cells and their potential as a therapeutic target. Semin Cell Dev Biol. 2020;107:63–71. 10.1016/j.semcdb.2020.05.001 32417217 PMC7554155

[111] Laco F , Woo TL, Zhong Q, et al. Unraveling the inconsistencies of cardiac differentiation efficiency induced by the GSK3β inhibitor CHIR99021 in human pluripotent stem cells. Stem Cell Reports. 2018;10:1851–1866. 10.1016/j.stemcr.2018.03.023 29706502 PMC5989659

[112] Watanabe K , Ueno M, Kamiya D, et al. A ROCK inhibitor permits survival of dissociated human embryonic stem cells. Nat Biotechnol. 2007;25:681–686. 10.1038/nbt1310 17529971

[113] Yu Z , Liu M, Fu P, et al. ROCK inhibition with Y27632 promotes the proliferation and cell cycle progression of cultured astrocyte from spinal cord. Neurochem Int. 2012;61:1114–1120. 10.1016/j.neuint.2012.08.003 22929997

[114] Jeong YJ , Hong Y, Yoon YJ, et al. Chemical reprogramming culture for the expansion of salivary gland epithelial basal progenitor cells. Stem Cell Res Ther. 2025;16:187. 10.1186/s13287-025-04295-5 40251601 PMC12008940

[115] Ninomiya I , Koyama A, Otsu Y, et al. Regeneration of the cerebral cortex by direct chemical reprogramming of macrophages into neuronal cells in acute ischemic stroke. Front Cell Neurosci. 2023;17:1225504. 10.3389/fncel.2023.1225504 37636590 PMC10457112

[116] Xiang J , Chen H, Zhang H, et al. Restoring sweat gland function in mice using regenerative sweat gland cells derived from chemically reprogrammed human epidermal keratinocytes. Sci Bull (Beijing). 2024;69:3908–3924. 10.1016/j.scib.2024.11.003 39550273

[117] Tan Z , Qin S, Liu H, et al. Small molecules reprogram reactive astrocytes into neuronal cells in the injured adult spinal cord. J Adv Res. 2024;59:111–127. 10.1016/j.jare.2023.06.013 37380102 PMC11081968

[118] Bai Y , Yang Z, Xu X, et al. Direct chemical induction of hepatocyte-like cells with capacity for liver repopulation. Hepatology (Baltimore, Md.). 2023;77:1550–1565. 10.1002/hep.32686 35881538

[119] Kannappan S , Kim Y, De D, et al. In vivo brown adipogenic reprogramming induced by a small molecule cocktail. Biomaterials. 2026;324:123463. 10.1016/j.biomaterials.2025.123463 40494021

[120] Moel M , Harinath G, Lee V, et al. Influence of rapamycin on safety and healthspan metrics after one year: PEARL trial results. Aging (Albany NY). 2025;17:908–936. 10.18632/aging.206235 40188830 PMC12074816

[121] Chung CL , Lawrence I, Hoffman M, et al. Topical rapamycin reduces markers of senescence and aging in human skin: an exploratory, prospective, randomized trial. Geroscience. 2019;41:861–869. 10.1007/s11357-019-00113-y 31761958 PMC6925069

[122] Vreones M , Mustapic M, Moaddel R, et al. Oral nicotinamide riboside raises NAD+ and lowers biomarkers of neurodegenerative pathology in plasma extracellular vesicles enriched for neuronal origin. Aging Cell. 2023;22:e13754. 10.1111/acel.13754 36515353 PMC9835564

[123] Yi L , Maier AB, Tao R, et al. The efficacy and safety of β-nicotinamide mononucleotide (NMN) supplementation in healthy middle-aged adults: a randomized, multicenter, double-blind, placebo-controlled, parallel-group, dose-dependent clinical trial. Geroscience. 2023;45:29–43. 10.1007/s11357-022-00705-1 36482258 PMC9735188

[124] Wen S , Zheng R, Cai C, et al. Chemical-based epigenetic reprogramming to advance pluripotency and totipotency. Nat Chem Biol. 2025;21:635–647. 10.1038/s41589-025-01874-8 40251434

[125] Schoenfeldt L , Paine PT, Picó S, et al. Chemical reprogramming ameliorates cellular hallmarks of aging and extends lifespan. EMBO Mol Med. 2025;17:2071–2094. 10.1038/s44321-025-00265-9 40588563 PMC12340157

[126] Kiourtis C , Terradas-Terradas M, Gee LM, et al. Hepatocellular senescence induces multi-organ senescence and dysfunction via TGFβ. Nat Cell Biol. 2024;26:2075–2083. 10.1038/s41556-024-01543-3 39537753 PMC11628396

[127] Li Z , Ge W, Li Y, et al. Valproic acid enhance reprogramming of bactrian camel cells through promoting the expression of endogenous gene c-myc and the process of angiogenesis. Int J Stem Cells. 2021;14:191–202. 10.15283/ijsc20213 33632993 PMC8138656

[128] Hu W , Qiu B, Guan W, et al. Direct conversion of normal and Alzheimer’s disease human fibroblasts into neuronal cells by small molecules. Cell Stem Cell. 2015;17:204–212. 10.1016/j.stem.2015.07.006 26253202

[129] Yin JC , Zhang L, Ma NX, et al. Chemical conversion of human fetal astrocytes into neurons through modulation of multiple signaling pathways. Stem Cell Reports. 2019;12:488–501. 10.1016/j.stemcr.2019.01.003 30745031 PMC6409415

[130] Parras A , Vílchez-Acosta A, Desdín-Micó G, et al. In vivo reprogramming leads to premature death linked to hepatic and intestinal failure. Nat Aging. 2023;3:1509–1520. 10.1038/s43587-023-00528-5 38012287

[131] Sela M , Chen G, Kadosh H, et al. AI-validated brain targeted mRNA lipid nanoparticles with neuronal tropism. ACS Nano. 2025;19:36106–36128. 10.1021/acsnano.4c15013 40957853 PMC12548354

[132] Zhou Y , Zhu F, Liu Y, et al. Blood-brain barrier-penetrating siRNA nanomedicine for Alzheimer’s disease therapy. Sci Adv. 2020;6: 10.1126/sciadv.abc7031 PMC7546706 33036977

[133] Lovell-Badge R , Anthony E, Barker RA, et al. ISSCR guidelines for stem cell research and clinical translation: the 2021 update. Stem Cell Reports. 2021;16:1398–1408. 10.1016/j.stemcr.2021.05.012 34048692 PMC8190668

