# Supplementary material for: Molecular time machines unleashed: small-molecule-driven reprogramming to reverse the senescence
Source: Stem Cells Transl Med. 2026 Jan 12;15(1):szaf069. doi: 10.1093/stcltm/szaf069 (PMC12798543; doi:10.1093/stcltm/szaf069)
Supplement: szaf069_Supplementary_Data [file szaf069_supplementary_data.docx]

**Molecular Time Machines Unleashed: Small Molecule-Driven Reprogramming to Reverse the** **Senescence**

Chunyin Tang^1,3^, Zhen Zhang^4^, Chunsong Yang^1,3^, Luxin Li^4^, Jie Li^1^, Xuejiao Cheng^1^, Wei Zhou^1^, Yunzhu Lin^1,3^*, Linan Zeng^1,2,3^^,^^5,6^* and Lingli Zhang^1,2,3,5,6^*

**Running head:** Chemical reprogramming to fight aging

^1^Department of Pharmacy/Evidence-Based Pharmacy Center, West China Second University Hospital, Sichuan University; Children's Medicine Key Laboratory of Sichuan Province, China

^2^NMPA Key Laboratory for Technical Research on Drug Products In Vitro and In Vivo Correlation, China

^3^Key Laboratory of Birth Defects and Related Diseases of Women and Children, Sichuan University, Ministry of Education, China

^4^Heilongjiang Key Laboratory of Anti-Fibrosis Biotherapy, Mudanjiang Medical University, Mudanjiang, China

^5^Chinese Evidence-based Medicine Center, West China Hospital, Sichuan University, China

^6^West China Biomedical Big Data Center, West China Hospital, Sichuan University, China

*Correspondence: Yunzhu Lin, PhD, Department of Pharmacy/Evidence-Based Pharmacy Center, West China Second University Hospital, Sichuan University, China E-mail: linyunzhu99@scu.edu.cn, tel: +86-028-85503059; Linan Zeng, PhD, Department of Pharmacy/Evidence-Based Pharmacy Center, West China Second University Hospital, Sichuan University, China E-mail: [zengl15@mcmaster.ca](mailto:zengl15@mcmaster.ca), tel: +86-028-85503059; or Lingli Zhang, PhD, Department of Pharmacy/Evidence-Based Pharmacy Center, West China Second University Hospital, Sichuan University, China. E-mail: [zhanglingli@scu.edu.cn,](mailto:zhanglingli@scu.edu.cn,) tel: +86-028-85503059.

**Supplementary Information**

**
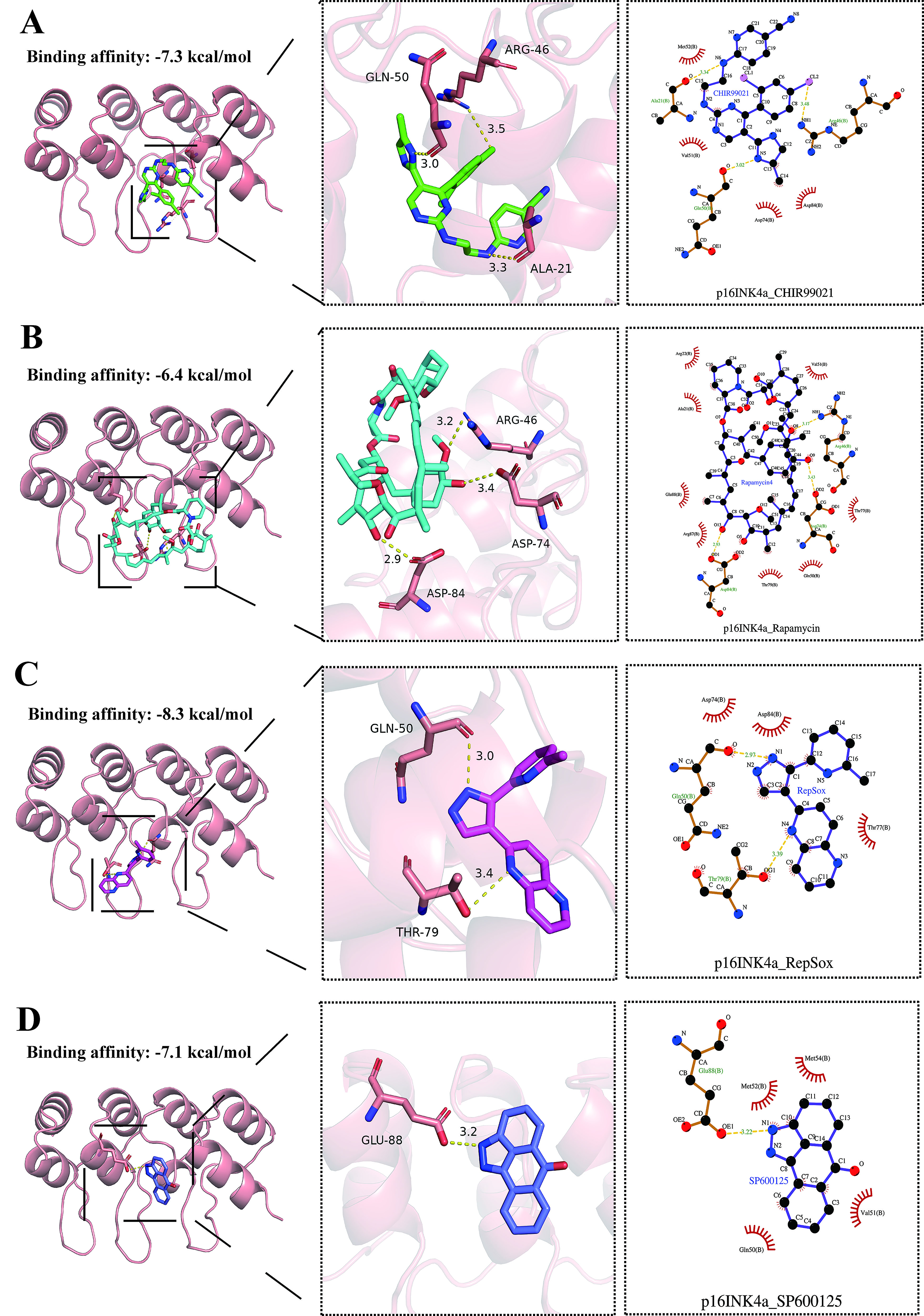
**

**Supplementary figure 1**. Details of GSK-3β inhibitor, mTOR inhibitor, TGF-β inhibitor and JNK inhibitor with p16INK4a (PDB: 7OZT) interaction. (A) Interactions (3D and 2D) of GSK-3β inhibitor CHIR99021 with p16INK4a (hydrogen bonds: ALA21, ARG46, and GLN50). (B) Interactions (3D and 2D) of mTOR inhibitor Rapamycin with p16INK4a (hydrogen bonds: ARG46, ASP74, and ASP84). (C) Interactions (3D and 2D) of TGF-β inhibitor RepSox with p16INK4a (hydrogen bonds: GLN50 and THR79). (D) Interactions (3D and 2D) of JNK inhibitor SP600125 with p16INK4a (hydrogen bonds: GLU88).

**Supplementary table 1**. Cell reprogramming technologies: reversing aging.

| **Terms** | **Merits** | **Deficits** | **Application** |
| --- | --- | --- | --- |
| SCNT | High cell totipotency and stable efficiency etc. | Ethical controversies, and resource dependence etc. | Basic Research ( e.g., developmental Biology) and animal model |
| iPS (OSKM) | Fully pluripotent stem cells available etc. | Carcinogenic risk, and episodic memory residues etc. | Disease modeling and drug screening |
| Small molecule cocktails***** | High security and low cost etc. | Low efficiency and long lead times etc. | Avoiding gene integration risks, suitable for clinical applications |

**Supplementary table 2**. Clinical trials of certain small molecules inducing cell fate

for rejuvenation.

| **No.** | **Drug delivery** | **Dose** | **Condition and age** | **Outcome measure** | **Interventional model** | **Country** |
| --- | --- | --- | --- | --- | --- | --- |
| NCT04488601 | Rapamycin,orally | 5 mg/week or 10 mg/week | Aging, 50-85 years | Visceral adiposity, blood biomarkers, lean tissue and bone mineral content | Parallel assignment | United States |
| [NCT03103893](http://clinicaltrials.gov/show/NCT03103893) | Rapamycin, topical cream | 10 μM | Dermal atrophy, 40-100 years | The level of p16^INK4A^ protein and  additional markers of senescence such as p21, p53 and collagen | Single group assignment | United States |
| NCT04823260 | NMN, orally | 300, 600, or 900 mg/ day | Aging, 40-65 years | Blood NAD concentration, blood biological age,insulin resistance and subjective general health assessment | Parallel assignment | India |
| NCT06208527 | NR, orally | A total of 2000 mg NR is administered daily for 1 year | Frail Elderly Syndrome and aging,  75 years and older | Clinical evaluations, including actigraphy and questionnaires，cognitive assessments，magnetic resonance imaging (MRI) and positron emission tomography (FDG-PET) scanning | Parallel assignment | Norway |
| NCT02921659 | NR, orally | 500 mg, 2x/day for 6 weeks | Aging, 55 to 79 years | NAD+ levels, levels of Aβ42, pJNK, and pERK1/2 (kinases involved in insulin resistance and neuroinflammatory pathways) | Crossover assignment | United States |
| NCT05706389 | α-Ketoglutarate, orally | 1 g/day | Aging, 40 to 60 years | The fasting blood sample, fasting triglycerides, high-density lipoprotein, glucose, and liver profile | Parallel assignment | Singapore |
| NCT06794255 | Vitamin C, orally | 500mg with water, twice a day for 12 consecutive months | Aging, 55-65 years (for the experimental and control groups) or 18-70 years | DNA methylation composite clock, blood cell transcriptomics, proteomics, metabolomics and gut microbiome in fecal sample | Parallel assignment | China |
| NCT01729598 | Valproic Acid, orally | 250 mg or 500mg by mouth twice daily. | AD, 65 to 90 years | Change in cerebrospinal fluid amyloid levels,change in cerebrospinal fluid tau levels, cognitive tests and change in cerebrospinal fluid clusterin levels | Parallel assignment | United States |
| NCT00974480 | Tretinoin, topical cream | 0.025% cream was applied twice a day every day, morning and evening for 24 weeks. | Skin aging, 45 to 55 years | Changes from baseline over time in skin aging as measured with the photonumeric scale, trans-epidermal Water Loss, skin hydration, skin elasticity and clinical skin evaluation etc | Parallel assignment | Canada |
